# Experimental and DFT Approaches to Physico-Chemical Properties of Bioactive Resveratrol Analogues

**DOI:** 10.3390/molecules29225481

**Published:** 2024-11-20

**Authors:** Borislav Kovačević, Ivana Šagud, Katarina Marija Drmić, Milena Mlakić, Irena Škorić, Sandra Babić

**Affiliations:** 1 Group for Computational Life Sciences, Division of Physical Chemistry, Ruđer Bošković Institute, Bijenička cesta 54, HR-10 000 Zagreb, Croatia; borislav.kovacevic@irb.hr; 2 Croatian Agency for Medicinal Products and Medical Devices, Ksaverska cesta 4, HR-10 000 Zagreb, Croatia; ivana.sagud@halmed.hr; 3 Department of Analytical Chemistry, Faculty of Chemical Engineering and Technology, University of Zagreb, Marulićev trg 19, HR-10 000 Zagreb, Croatia; kdrmic@fkit.unizg.hr; 4 Department of Organic Chemistry, Faculty of Chemical Engineering and Technology, University of Zagreb, Marulićev trg 19, HR-10 000 Zagreb, Croatia; mdragojev@fkit.unizg.hr

**Keywords:** ADMET, DFT study, hydrogen bond acidity, p*K*_a_ values, resveratrol analogues

## Abstract

Acetylcholinesterase and butyrylcholinesterase are two related enzymes that represent pharmacologically suitable targets in neurodegenerative disorders, given their physiological roles in the body. The treatment of neurodegenerative disorders currently includes common reversible cholinesterase inhibitors. Resveratrol analogues, as the molecules in focus, have shown the very strong inhibition potential of cholinesterases. In this research, experimental and DFT approaches for their p*K*_a_ value determination were carried out knowing that p*K*_a_ is very important for predicting the ADMET properties of the potentially bioactive molecules and their behavior in the environment. An in silico study was used to calculate more indicators about the absorption and distribution in the human body. Among the investigated compounds, the weakest acid was experimentally detected and confirmed using three computational models. Additionally performed calculations provided access to the potential of each resveratrol analogue to engage in both π-π stacking and hydrogen bond interactions in the active site of the enzyme crucial for the stability of the ligand–enzyme complex.

## 1. Introduction

**Figure 1:**
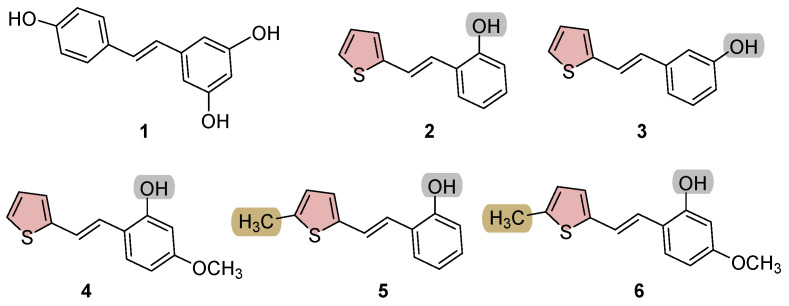
Structures of the *trans*-resveratrol (**1**) and the investigated biologically active resveratrol analogues **2**–**6**.

Inspired by naturally occurring *trans*-resveratrol (structure **1**, Figure 1) and its resourceful biological activity, especially in neurodegenerative disorders [1,2,3,4,5,6,7,8,9,10,11,12,13,14,15,16,17], in our previous research, we designed and synthesized its 22 heterocyclic analogues as potential cholinesterase inhibitors [18,19], with an OH group placed at different positions on the aryl ring. It is known that the treatment of Alzheimer’s disease is exclusively symptomatic and affects mainly the alleviation of symptoms and is based mainly on increasing the concentration of acetylcholine (ACh) by inhibiting cholinesterase enzymes. Biological testing on resveratrol analogues was conducted and five of them (structures **2**–**6**, Figure 1) exhibited significantly enhanced butyrylcholinesterase (BChE) inhibitory (2.4–6.2 μM) and antioxidant activity (DPPH, 0.51–82.3 μM) compared to established standards such as galantamine (7.9 μM) or resveratrol (DPPH, 74.0 μM [20]). To gain insights into their molecular interactions, molecular docking studies were performed of those five selected ligands into BChE, and molecular dynamics (MD) simulations validated the resulting complexes’ stability. Indeed, the best results in the described research were shown by heteroaromatic resveratrol analogues that had the OH group placed mostly in the *ortho* or *meta* position on the aryl ring (compounds **2**–**6**, Figure 1). Lhasa software (Nexus v.2.5.2 (Build 5, Jul 2022), Derek Nexus v.6.2.1; Sarah Nexus v.3.2.1) was employed to identify their potential mutagenicity. It is worth noting that none of these derivatives showed a positive result.

By introducing new substituents on the aromatic ring of the resveratrol-like scaffold [21], we analyzed which new positions and substituents could contribute the most to biological activity. However, the main intention was to see if this second series of compounds [21] would show any better results compared to the previous research [18,19]; it was not the case. Although the enzyme BChE exhibited again greater sensitivity to these heteroaromatic resveratrol analogues than AChE, the best compounds achieved the BChE inhibition with IC_50_ values from 22.9 to 39.7 μM. From the results presented so far, it was concluded that compounds **2**–**6** (Figure 1) from the first series represent the best potential candidates as new therapeutics for neurological disorders so they can be incorporated into chemical libraries used in high-throughput screening and drug development.

When investigating a molecule as an active pharmaceutical ingredient, it is crucial to take into consideration how well this molecule will perform in all aspects of ADME(T) [22]. For small molecules that are in very early phases of investigation, one of the first steps to consider and possibly optimize is solubility in water, which is directly connected to dissociation in water. The acid–base dissociation constant (p*K*_a_) is a physico-chemical parameter that can serve as a key early indicator of the bioavailability of the drug substance [23,24]. However, as the p*K*_a_ is not only an indicator of the mentioned solubility but also the oral absorption, distribution, and pharmacokinetics of the drug, thus, having an in-depth knowledge is crucial for the early development of the drug substance as well as all future steps in the development of the drug product. Understanding the p*K*_a_ helps in determining the ionization state of a drug at different physiological pH levels. If the p*K*_a_ of a drug is close to the physiological pH (around 7.4), the drug will be mostly ionized in the body, affecting its ability to cross cell membranes or bind to target sites. If it is far from the physiological pH (meaning above the 7.4 level), the drug is likely to be in a non-ionized form, which is generally more lipophilic and can easily penetrate cell membranes [25]. It is important to note that, in early development, the drug substance p*K*_a_ can be optimized by functional group changes, and, later in formulation optimizations with lead molecules, the choice of excipients will be profoundly affected by the drug substance p*K*_a_.

Taking into account the encouraging experimental results obtained by testing the inhibition potential of the cholinesterase enzyme [26,27,28,29,30,31], and the importance of the compound’s physico-chemical characteristics in the assessment of the bioavailability as the potential drug substance, in this research, experimental and DFT approaches to p*K*_a_ determination for bioactive resveratrol analogues **2**–**6** (Figure 1) were carried out. When doing so, it is kept in mind that the determination of p*K*_a_ is as important for predicting the ADMET properties of the potentially biologically active molecules as it is for their behavior in the environment. From all the above-mentioned reasons, in this work, we additionally performed calculations to quantitatively assess the potential of each resveratrol analogue to engage in both π-π stacking and hydrogen bond interactions in the active site of the enzyme crucial for the stability of the ligand–enzyme complex.

## 2. Results and Discussion

### 2.1. Prediction of pK_a_ Values for Resveratrol Analogues ***2***–***6*** and In Silico Study of Their Absorption and Distribution in the Human Body

As stated, the acid-base dissociation constant (p*K*_a_) is a physico-chemical parameter that can serve as a key early indicator of the bioavailability of the drug substance. The p*K*_a_ of a drug influences lipophilicity, solubility, potential for protein binding and permeability, which then affects pharmacokinetics (absorption, distribution, metabolism, and excretion). This relevant indicator can be investigated very early on primarily with in silico tools. One of the best and mostly available software options is the “MarvinSketch” [32]. For this work, the utilization of this tool was the first step in the determination of the future investigation into the p*K*_a_ values of the investigated resveratrol analogues **2**–**6** (Figure 1). The calculated values are presented in Table 1.

These values gave an insight into which groups could have an impact on the p*K*_a_ of the studied molecules. With more in-depth theoretical calculations performed later, some of the insights were explained, and this will be further elaborated in detail later in this work. As understanding the p*K*_a_ helps in determining the ionization state of a drug at different physiological pH levels, these compounds can be placed in the group that probably exists mostly in the non-ionized form. These compounds are generally more lipophilic and can easily penetrate cell membranes. But, nevertheless, as the compounds do have the OH (ionizable group), in general, such groups in molecules can be used to modify overall polarity, which in turn controls some physicochemical properties (aqueous solubility or hydrophilicity), and these functional groups can interact with target macromolecules.

The next preliminary investigative step was to see how these molecules behave in the in silico study, which calculates more indicators about the absorption and distribution in the human body. For this study, a free in silico tool [33] was used in order to screen the candidates. The results are presented in Table 2 below.

The solubility in water (linked to dissolution for the orally administered drug product) and the permeability and absorption in the intestines and or skin (depending on the route of administration) are very good indicators of the future absorption of the active substance. The solubility of all of these compounds is low, but this is not a rare occurrence with active drug substances, and it can be modified by appropriate drug product formulations. Moderate permeability and a high HIA (human intestinal absorption) are indicators that they can be good candidates for an oral route of administration even if the solubility in water is low. As for the potential for the molecule to cross the CNS barrier, these molecules all have a Log BB above the minimum of 0.3, which would indicate a good distribution to the brain, even though there is no potential for P-glycoprotein inhibition.

### 2.2. Experimental Approach to pK_a_ Determination

**Figure 2:**
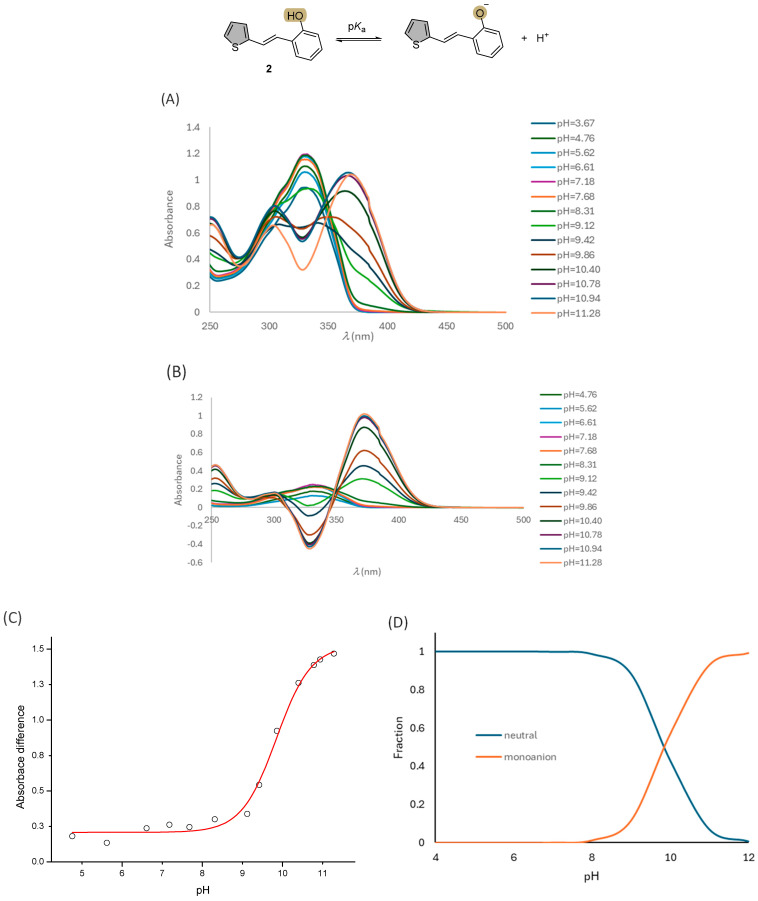
Spectral data and p*K*_a_ determination for compound **2**: (**A**) corrected spectra (*A* = 0 for *λ* = 500 nm), (**B**) spectral difference plot (maximum negative deviation at 328 nm and maximum positive deviation at 372 nm), (**C**) total absorbance difference vs. pH, and (**D**) distribution of species as a function of pH.

**Figure 3:**
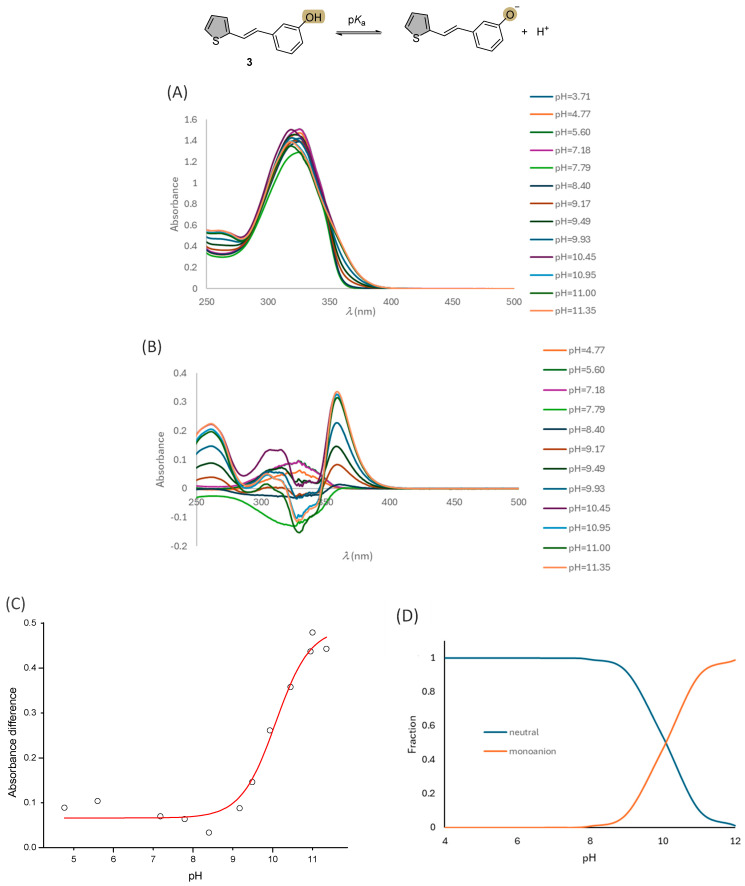
Spectral data and p*K*_a_ determination for compound **3**: (**A**) corrected spectra (*A* = 0 for *λ* = 500 nm), (**B**) spectral difference plot (maximum negative deviation at 330 nm and maximum positive deviation at 359 nm), (**C**) total absorbance difference vs. pH, and (**D**) distribution of species as a function of pH.

For the successful determination of p*K*_a_ values using the UV/vis spectrometric method, the UV/vis absorbance of the sample must change as a function of ionization. Figure 1 shows the structures of the investigated resveratrol analogues. Each resveratrol analogue has a single ionizable group, acidic phenol attached to a heterocycle via an allyl bond representing a strong chromophore. The ionization of resveratrol analogue molecules (the loss of H^+^) changes the arrangement of electrons in the chromophore, and, as a result, the UV/vis spectra of ionized and neutral species are different. This can be clearly seen in Figure 2A, which shows the UV/vis spectrum for compound 2, as an example. For the other investigated compounds, the spectra and results of data analysis are presented in the Supporting Information (Figures S1–S3). All compounds showed UV/vis spectra that passed through the isosbestic point, indicating a smooth transition from one species to another within the pH range involved in p*K*_a_ determination. At lower pH values, the molecules are neutral with an absorption maximum between 311 nm and 343 nm. At the highest pH value, only deprotonated species are present with two absorption maxima, one at a wavelength around 370 nm and the other around 305 nm. The exception is compound **3**, which does not show significant changes in the spectrum with a change in pH values (Figure 3A). Data analysis for the determination of ionization constants by spectral analysis applied in this study is described by Tomsho et al. [34] and Ríos Martínez and Dardonville [35]. The results of data analysis on investigated resveratrol analogues, spectral difference, and total absorbance difference plots are shown on Figure 2B,C, Figure 3B,C and Figures S1B,C, S2B,C and S3B,C.

The results of the p*K*_a_ determinations for all substances are summarized in Table 3. The results of repeated measurements show satisfactory precision with scatter values less than or equal to 0.06 for all compounds [36].

Among the investigated compounds, compound **4** is the strongest acid, stronger by almost one p*K*_a_ unit than compound **3**, which is the weakest acid. The p*K*_a_ values are evenly distributed between p*K*_a_ = 9.17 (compound **4**) and 10.07 (compound **3**), with a step of approximately 0.2 p*K*_a_ units and the following order of decreasing acid strength: **4** > **5** > **6** > **2** > **3**.

### 2.3. Computational Approach to pK_a_ Determination

To verify experimental data and to rationalize differences in p*K*_a_ values between resveratrol analogues (**2**–**6**), we have calculated p*K*_a_ values using three computational models. They are denoted as follows: M1 = SMD-B3LYP-D3/6-311+G(3df,2p), M2 = SMD-ωB97XD/6-311+G(3df,2p), and M3 = SMD-M062X/6-311+G(3df,2p). Results are summarized in Table 4. Gas phase acidity is marked by Δ*G*_acid_ and is calculated by ωB97XD/6-311+G(3df,2p) model (MII) only.

**Figure 4:**
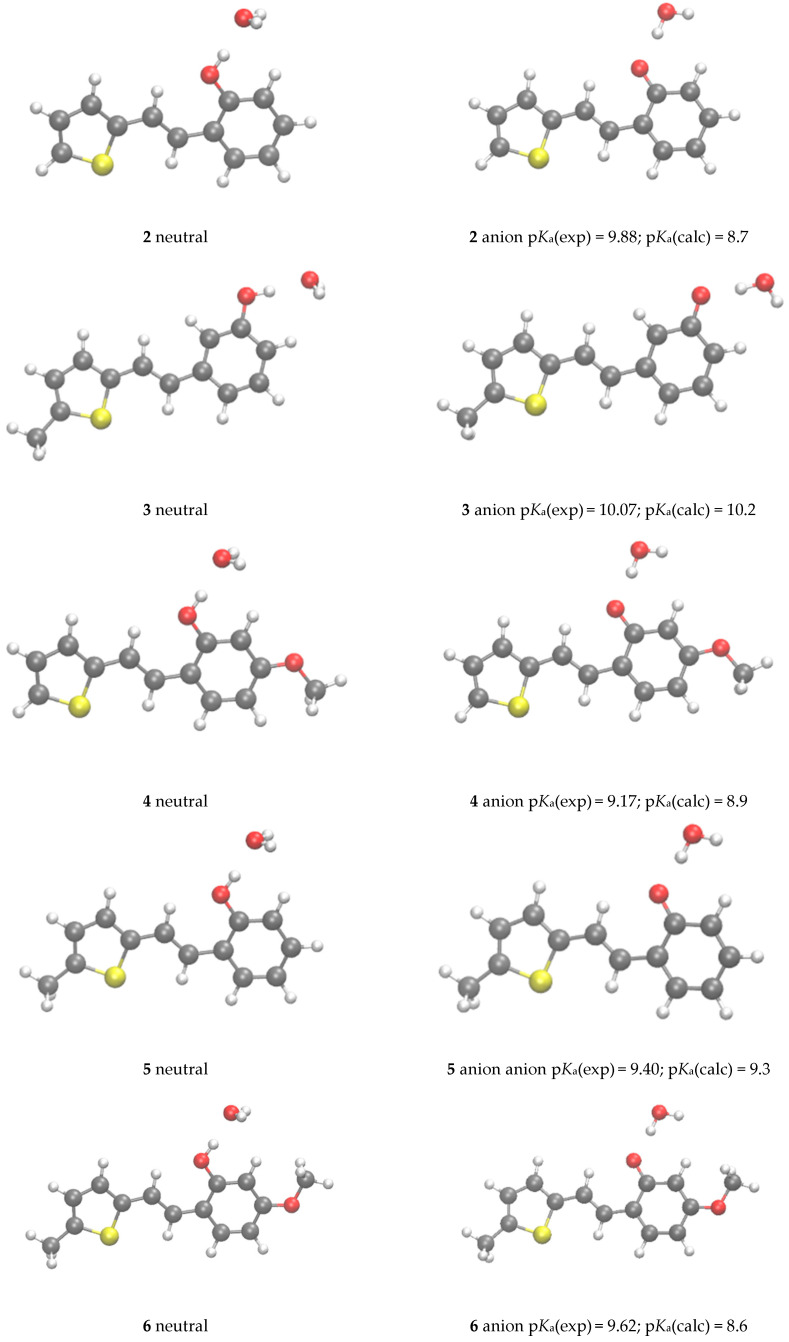
Calculated structures for compounds **2**–**6** and their corresponding anions along with their p*K*_a_ values (theoretical and experimental). The presented theoretical p*K*_a_ values were obtained using model M2.

Comparing the calculated p*K*_a_ values with the experimental ones reveals that the calculated p*K*_a_s are systematically lower, with model M2 showing the best agreement with the experimental data. This is evident from the mean absolute error (MAE), which is 0.4 for model M2, compared to 1.1 and 1.0 for models M1 and M3, respectively. Figure 4 displays both a comparison of the experimental and theoretical p*K*_a_ values obtained using the M2 model, as well as the optimized structures of the neutral and deprotonated species, each solvated by a single water molecule. It should be noted, however, that the variation in experimental p*K*_a_ values between compounds **2**–**6** is very small, ranging by only 0.9 units, which is relatively close to M2 MAE. Therefore, one should be cautious in drawing far-reaching conclusions based on such small differences.

**Scheme 1:**
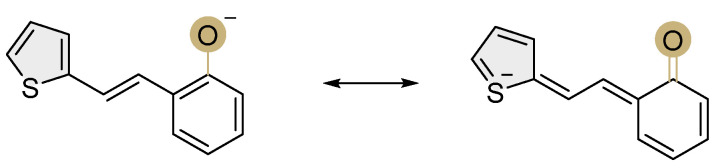
Resonance structures in *ortho*-substituted resveratrol analogues.

Nonetheless, both experimental data and all three computational models consistently predict that compound **3** is the weakest acid in the series. Compound **3** is the only compound in which the ethenyl thiophene group is in the *meta* position relative to the OH group, while, in all other compounds, the group is in the *ortho* position. A plausible explanation for the lower acidity of compound **3** is that the *ortho*-substituted ethenyl thiophene group in compounds **2** and **4**–**6** may stabilize the phenolate anion through resonance/mesomeric effects (Scheme 1).

**Scheme 2:**
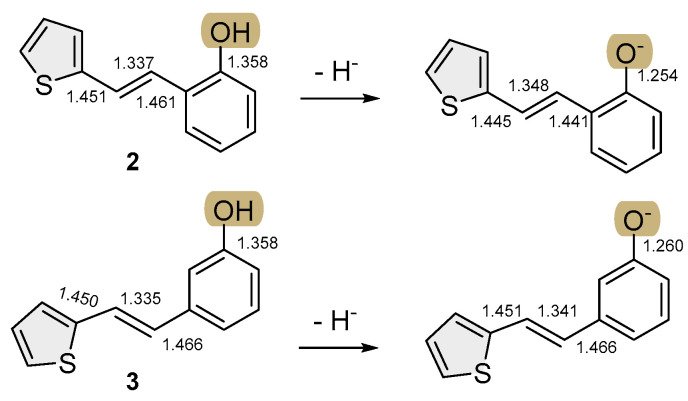
Change in selected bond lengths upon deprotonation in *ortho*- and *meta*-substituted resveratrol analogues. Change in all bond distances is presented in Table S1.

The resonance shown above which is not present in compound **3** is evident by the inspection of changes in selected bond distances upon deprotonation (Scheme 2).

Consequently, the higher p*K*_a_ of compound **3** should be attributed to its inherently lower acidity in the gas phase, which is confirmed by its highest Δ*G*_acid_ value (Table 4).

It is more difficult, however, to rationalize the other p*K*_a_ differences. For example, computational results obtained with model M2 suggest that compound **6** is more acidic than compound **5**, which is inconsistent with the experimental measurements. Considering that the only structural difference between compounds **5** and **6** is the methoxy group in the *meta* position (relative to the OH group) in **6**, one might expect compound **6** to be more acidic. This expectation arises from the fact that *meta*-methoxyphenol (experimental p*K*_a_^exp^ = 9.6) is more acidic than phenol (p*K*_a_^exp^ = 9.98) due to the -I inductive effect, while the mesomeric effect is negligible.

Gas phase acidity (Δ*G*_acid_) calculations also indicate that compound **6** is more acidic than compound **5** by 1.8 kcal mol^−1^; therefore, the discrepancy between experimental and computational results is not entirely clear. The similar ambiguity holds if we compare **2** and **4**. Again, the difference between these two is *meta* methoxy group in **4**; however, in this case, the computational results (M2) give that **4** is less acidic than **2** (by 0.2 units), whereas the experimental results indicate vice versa (by 0.7 units). Assuming the experimental results are accurate, the error in the computational acidity order of the compounds could stem from a miscalculation of the inherent (gas-phase) acidity or inaccuracies in the solvation model. By comparing the acidity order in solution, as predicted by model M2, with the gas-phase Δ*G*_acid_ values obtained using the same functional and basis set, we observe that the acidity order is identical in both cases (**3** < **5** < **4** < **2** < **6**). Therefore, we can conclude that the computational error likely arises from a miscalculation of inherent basicity, rather than from inaccuracies in the solvation model. Presumably, there is an interplay between mesomeric and inductive effects in **2**–**6** that is quite subtle and thus very challenging to quantify accurately. To address these effects more accurately, more sophisticated but also significantly more expensive computational methods, such as CBS-QB3, CBS-APNO, G4, or CCSD(T), should be used.

In our earlier studies [18,21], we demonstrated that these resveratrol analogues form stable non-covalent complexes with cholinesterases (ChE), leading to their inhibition. The active site of ChEs is rich in aromatic residues, facilitating the stabilizing interactions between the studied molecules and these residues, primarily through π-π stacking. Additionally, the presence of serine in the active site allows for the possibility of hydrogen bond formation between the resveratrol analogues and the serine side chain. In this work, we additionally performed calculations to quantitatively assess the potential of each resveratrol analogue to engage in both π-π stacking and hydrogen bond interactions. To quantify the π-π stacking ability, we calculated the enthalpy of complexation (∆*H*_π-π)_ between resveratrol analogues **2**–**6** and benzene as a probe. It should be noted that for all π-π complexes between **2** and **6** and benzene, the parallel displaced structure was the most stable, and ∆*H*_π-π_ for this structure only is shown in Table 5.

To estimate the hydrogen-bond-forming ability of compounds **2**–**6**, we calculated the hydrogen bond enthalpy (Δ*H*_HB_) for their complexes with methanol used as a probe. Both ∆*H*_π-π_ and ∆*H*_HB_ were calculated utilizing M2 in diethyl ether, as its dielectric constant resembles the environment of the enzyme active site [37].

Interestingly, compound **3** exhibited the lowest ∆*H*_π-π_ and ∆*H*_HB_ values, which aligns with its experimentally observed lowest inhibition potential.

**Figure 5:**
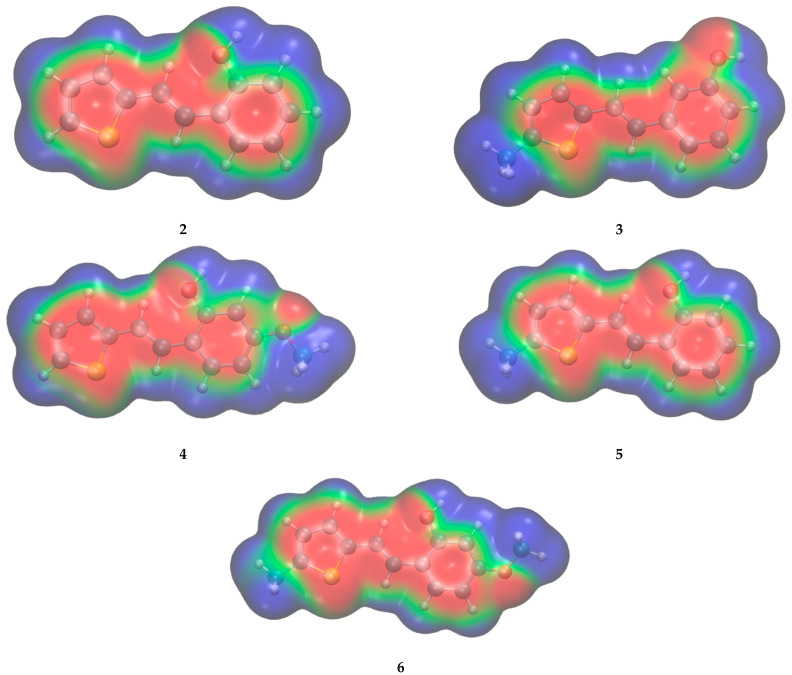
Electrostatic potential map for compounds **2**–**6**. Electrostatic surface potentials are colored blue and red for positive and negative charges, respectively, and green represents neutral regions.

To further elucidate how compounds **2**–**6** might interact with their biological target, we calculated the electrostatic potential map, as shown in Figure 5.

The calculated electrostatic potential maps, which illustrate the charge distribution within compounds **2**–**6** (Figure 5), show that the aromatic rings of phenol and thiophene connected by an ethylene bridge along with an oxygen atom of hydroxy and methoxy groups are the most negatively charged regions of the scaffold. Conversely, the hydrogens on the periphery of the molecule, as well as the methyl group(s), are the most positively charged, as expected.

## 3. Materials and Methods

### 3.1. Materials

Resveratrol analogues **2**–**6** were synthesized, according to the procedure described in [18], as mixtures of two configurational isomers in different ratios by the Wittig reaction using triphenylphosphonium salts and various benzaldehydes. The obtained mixtures of isomers were purified by extraction and column chromatography, where the petroleum ether/ether (PE/E) solvent system of variable proportions was used to isolate the targeted compounds bearing *trans*-configuration in the structure. All of the synthesized new phenolic stilbenes, resveratrol analogs **2**–**6**, have been fully proven by NMR, UV, and MS, and their purity was confirmed by HRMS analyses. All compounds are >95% pure by HPLC analysis. ^1^H and ^13^C NMR spectra were recorded on a Bruker (Billerica, MA, USA) Avance spectrometer operating at a frequency of 600 MHz for ^1^H nuclei and a frequency of 150 MHz for ^13^C nuclei. Deuterated chloroform—CDCl_3_—and deuterated methanol—CD_3_OD—were used to dissolve compounds, and tetramethylsilane—TMS—was used as a standard for recording NMR spectra. Chemical shifts are expressed in ppm (parts per million), and the following symbols were used to characterize the signals: s—singlet, d—doublet, dd—doublet of doublets, dt—doublet of triplets, t—triplet, and m—multiplet. HRMS analyses to confirm the samples’ purity were carried out on a mass spectrometer (MALDI TOF/TOF analyzer) equipped with a Nd:YAG laser operating at 355 nm with an adjustment speed of 200 Hz.

Stock solutions of resveratrol analogues were prepared at a concentration of 5 mM in p.a. methanol (Gram-Mol, Zagreb, Croatia). Working solutions were prepared by 100-fold dilution of stock solutions with appropriate buffer solutions. In this way, the concentrations of resveratrol analogues in the working solutions were 0.05 mM, and the volume fraction of methanol was 1%.

Chemicals used for preparation of buffers were sodium acetate (Alkaloid, Skopje, Macedonia), potassium dihydrogenphosphate (Lach-ner, Neratovice, Czech Republic), sodium hydrogencarbonate (Kemika, Zagreb, Croatia), and dipotassium hydrogenphosphate (Fisher Chemical, Loughborough, UK). Sodium hydroxide was supplied by Gram-mol (Zagreb, Croatia), and hydrochloric acid was supplied by VWR Chemicals (Leuven, Belgium). All chemicals used were of analytical grade and used as received. For the preparation of buffer solutions for spectrophotometric measurements, stock solutions of 0.1 M sodium acetate, 0.05 M potassium dihydrogen phosphate, 0.05 M sodium hydrogen carbonate, and 0.05 M dipotassium hydrogen phosphate were used. Sodium acetate/acetic acid buffer was used for pH range 3.5−5.0, KH_2_PO_4_/K_2_HPO_4_ for pH range 5.5–8.5, NaHCO_3_/H_2_CO_3_ for pH range 9.0−11.0, and K_2_HPO_4_/K_3_PO_4_ for pH range 11.0−11.5. All buffer solutions were prepared with the same ionic strength (*I* = 0.1 M) by adding KCl (Lach-ner, Neratovice, Czech Republic).

Deionized water was prepared using a Milli-Q water system (Millipore, Molsheim, France).

### 3.2. Spectroscopic Data for ***2***–***6***

*(E)-2-(2-(Thiophen-2-yl)vinyl)phenol* (**2**): 495 mg, 82% isolated; white powder, m. p. 126–127 °C; R*_f_* (PE/DCM (50%)) = 0.33; UV (ACN) *λ*_max_/nm (*ε*/dm^3^mol^−1^cm^−1^) 335 (27,412); ^1^H NMR (CDCl_3_, 600 MHz) *δ*/ppm: 7.46 (dd, *J* = 7.7, 1.6 Hz, 1H), 7.28 (dt, *J* = 16.1, 0.8 Hz, 1H), 7.20–7.16 (m, 2H), 7.15–7.10 (m, 1H), 7.07 (dt, *J* = 3.7, 0.9 Hz, 1H), 6.99 (dd, *J* = 5.1, 3.5 Hz, 1H), 6.93 (dt, *J* = 7.5, 1.2 Hz, 1H), 6.78 (dd, *J* = 8.1, 1.2 Hz, 1H), 4.97 (s, 1H); ^13^C NMR (CDCl_3_, 150 MHz) *δ*/ppm: 152.9, 143.3, 128.6, 127.6, 127.2, 126.0, 124.4, 124.3, 123.3, 122.7, 121.2, 115.9; MS (ESI) (*m/z*) (%, fragment): 203 (100); HRMS (*m/z*) for C_12_H_10_OS: [M + H]^+^_calculated_ = 202.0452, [M + H]^+^_measured_ = 202.0449.
*(E)-3-(2-(Thiophen-2-yl)vinyl)phenol* (**3**): 13 mg, 5% isolated; white powder; m. p. 86–88 °C; *R_f_* (PE/E (18%)) = 0.24; ^1^H NMR (CDCl_3_, 600 MHz) *δ*/ppm: 7.22–7.18 (m, 3H), 7.06 (d, *J* = 3.9 Hz, 1H), 7.04 (d, *J* = 7.9 Hz, 1H), 7.00 (dd, *J* = 5.4, 3.3 Hz, 1H), 6.94 (t, *J* = 2.1 Hz, 1H), 6.86 (d, *J* = 15.9 Hz, 1H), 6.72 (dd, *J* = 7.9, 2.5 Hz, 1H), 4.78 (s, 1H); ^13^C NMR (CDCl_3_, 150 MHz) *δ*/ppm: 155.8, 142.7, 138.7, 129.9, 127.8, 127.6, 126.3, 124.5, 122.3, 119.3, 114.7, 112.7; MS (ESI) (*m/z*) (%, fragment): 203 (100); HRMS (*m/z*) for C_12_H_10_OS: [M + H]^+^_calculated_ = 202.0452, [M + H]^+^ _measured_ = 202.0458.
*(E)-5-Methoxy-2-(2-(thiophen-2-yl)vinyl)phenol* (**4**): 60 mg, 40% isolated; yellow powder; m. p. 91–94 °C; *R_f_* (PE/E (60%)) = 0.35; UV (ACN) *λ*_max_/nm (*ε*/dm^3^mol^−1^cm^−1^) 336 (23,732), 244 (9367), 211 (15,320); ^1^H NMR (CDCl_3_, 600 MHz) *δ*/ppm: 7.36 (d, *J* = 8.7 Hz, 1H), 7.17–7.14 (m, 2H), 7.06 (d, *J* = 16.2 Hz, 1H), 7.03 (d, *J* = 3.4 Hz, 1H), 6.98 (dd, *J* = 4.9, 3.7 Hz, 1H), 6.51 (dd, *J* = 8.5, 2.6 Hz, 1H), 6.37 (d, *J* = 2.6 Hz, 1H), 5.03 (s, 1H), 3.79 (s, 3H); ^13^C NMR (CDCl_3_, 150 MHz) *δ*/ppm: 160.2, 153.9, 143.6, 128.2, 127.2, 125.4, 123.8, 122.6, 121.5, 117.3, 107.1, 101.9, 55.4; MS (ESI) (*m/z*) (%, fragment): 233 (100); HRMS (*m/z*) for C_13_H_12_O_2_S: [M + H]^+^_calculated_ = 232.0558, [M + H]^+^_measured_ = 232.0556.
*(E)-2-(2-(5-Methylthiophen-2-yl)vinyl)phenol* (**5**): 95 mg, 40% isolated; yellow powder; m. p. 88–92 °C; *R_f_* (PE/E (20%)) = 0.54; UV (ACN) *λ*_max_/nm (*ε*/dm^3^mol^−1^cm^−1^) 339 (26,378), 240 (10,925), 208 (19,311); ^1^H NMR (CDCl_3_, 600 MHz) *δ*/ppm: 7.44 (d, *J* = 7.3 Hz, 1H), 7.18 (d, *J* = 16.6 Hz, 1H), 7.11 (t, *J* = 7.9 Hz, 1H), 7.01 (d, *J* = 16.6 Hz, 1H), 6.92 (t, *J* = 7.6 Hz, 1H), 6.85 (d, *J* = 2.9 Hz, 1H), 6.78 (d, *J* = 7.9 Hz, 1H), 6.64–6.64 (m, 1H), 4.93 (s, 1H), 2.48 (s, 3H); ^13^C NMR (CDCl_3_, 150 MHz) *δ*/ppm: 152.8, 141.2, 139.3, 128.3, 127.1, 126.3, 125.7, 124.5 123.8, 121.4, 121.2, 115.9, 115.6; MS (ESI) (*m/z*) (%, fragment): 217 (100); HRMS (*m/z*) for C_13_H_12_OS: [M + H]^+^_calculated_ = 216.0609, [M + H]^+^_measured_ = 216.0607.
*(E)-5-Methoxy-2-(2-(5-methythiophen-2-yl)vinyl)phenol* (**6**): 15 mg, 16% isolated; yellow powder; m.p. 91–95 °C; *R_f_* (PE/E (60%)) = 0.45; ^1^H NMR (CDCl_3_, 600 MHz) *δ*/ppm: 7.33 (d, *J* = 8.4 Hz, 1H), 7.06 (d, *J* = 16.3 Hz, 1H), 6.91 (d, *J* = 16.3 Hz, 1H), 6.80 (d, *J* = 3.4 Hz, 1H), 6.62 (d, *J* = 3.1 Hz, 1H), 6.50 (dd, *J* = 8.7, 2.6 Hz, 1H), 6.37 (d, *J* = 2.5 Hz, 1H), 5.03 (s, 1H), 3.78 (s, 3H), 2.47 (s, 3H); ^13^C NMR (CDCl_3_, 150 MHz) *δ*/ppm: 160.0, 153.9, 141.5, 138.7, 128.0, 125.7, 121.9, 121.3, 117.5, 107.1, 101.9, 55.4, 15.6; MS (ESI) (*m/z*) (%, fragment): 245 (100); HRMS (*m/z*) for C_14_H_14_O_2_S: [M + H]^+^_calculated_ = 246.0715, [M + H]^+^_measured_ = 246.0712.

### 3.3. Instrumentations and Methods

The exact pH value of the prepared buffer solutions was measured with an S20 SevenEasy pH-meter (MettlerToledo, Greifenseea, Switzerland).

UV/visible spectra of resveratrol analogue working solutions were recorded using Lambda 35 UV/vis spectrometer (PerkinElmer, Walthama, MA, USA) in the wavelength range from 200 nm to 700 nm. The data analysis was carried out in the entire specified range. However, due to high absorbance at short wavelengths and zero absorbance at wavelengths greater than 500 nm, spectra in the range from 250 nm to 500 nm are shown in the figures for a clearer presentation. Working solutions of resveratrol analogues as well as blank solutions were placed in quartz cuvettes with 10 mm path length. Appropriate buffer solutions were used as a blank.

Data analysis included correction of the raw spectra (*A*(500 nm) = 0) and the calculation of the spectral difference, which were performed using Excel program. The spectral difference was determined between the spectra recorded at each pH and at the spectra recorded at the lowest pH. The wavelengths of maximum positive and negative deviations were determined from the spectral difference graph. The absolute values of the absorbance difference at the determined wavelengths were summed to obtain total absorbance difference. The dependence of the total absorbance difference on pH (spectral difference graph) gives a characteristic sigmoidal curve. In order to determine the p*K*_a_ values of investigated resveratrol analogues, the experimentally determined total absorbance difference values were fitted to Equation (1) [34,35] by non-linear regression (OriginPro, v.8.5).

(1) $$Total\;absorbace\;difference\;=\;\left[S_{T}\right]\frac{\varepsilon_{HA}-\varepsilon_{A^{-}}\cdot 10^{\left(pH-{pK}_{a}\right)}}{1+10^{\left(pH-{pK}_{a}\right)}}$$

where [S_T_] is the total substance concentration, and *ε_HA_* and *ε_A_^−^* represent the extinction coefficients of the acidic and basic form of the compound, respectively.

Measurements were performed using freshly prepared working solutions. For each resveratrol analogue and each pH, three consecutive measurements were made.

### 3.4. Computational Details

Schlegel at all [38] tested 175 different density functionals for calculation of p*K*_a_ of thiols. They found that with the B3LYP and ωB97XD functionals, the calculated pK_a_s are within one unit of the experimental values, whereas most other functionals used in this study substantially underestimate the p*K*_a_s. Furthermore, in the selection of the basis set, they demonstrated that polarization functions on the hydrogen atoms and diffuse functions on the heavier atoms are necessary. Therefore, the p*K*_a_ values of resveratrol analogues (**2**–**6**) in water were calculated using three of the most commonly used density functionals that are expected to yield reliable results: B3LYP-D3 [39,40], ωB97XD [41], and M062X [42] in combination with the 6-311+G(3df,2p) basis set. Solvent effects were modeled using the SMD implicit solvation model [43], with the inclusion of a single explicit water molecule to account for specific solute–solvent interactions. This approach with explicit solvent molecules was employed to enhance the accuracy of the solvation energy calculations and the resulting p*K*_a_ predictions [44]. It should be noted that in the study of Schelegel et al., as well as in some previous studies [25], on the calculation of p*K*_a_ for resveratrol analogs, the authors employed a method incorporating up to three water molecules in the first solvation shell. However, as pointed out in the paper by Zimányi et al. [25], the large number of possible configurations using more water molecules can lead to errors due to inaccurately determined arrangements of the water molecules relative to the solute.

p*K*_a_ values are calculated relative to experimental p*K*_a_ value of phenol (p*K*_a_ = 9.98) [45] using the following scheme

$${{\mathrm{AH}}^{q}}_{\left(\mathrm{solv}\right)}\;+\;{{\mathrm{Ref}}^{q-1}}_{\left(\mathrm{solv}\right)}\;\overset{\Delta G\mathrm{solv}}{\to }\;{A^{q-1}}_{\left(\mathrm{solv}\right)}\;+\;{{\mathrm{RefH}}^{q}}_{\left(\mathrm{solv}\right)}$$

where ΔG_solv_ = *G*^298^(A^q−1^)_solv_ + *G*^298^(RefH^q^)_(solv)_ − *G*^298^(AHq)_soln_ − *G*^298^(AH^q^)_(solv)_ and p*K*_a_ = ΔG_solv_/2.303RT. AH represents the acids being studied (resveratrols), while “Ref” denotes the reference acid with known p*K*_a_ value (phenol).

Geometries of the neutral and deprotonated forms of phenol and resveratrol analogues were fully optimized in solution. Their status as energy minima on the potential energy surface was verified by computing vibrational frequencies. To assess acidity in the gas phase, the Gibbs free-energy change (Δ*G*_acid_) was calculated for the gas-phase deprotonation reaction:

AH → A^−^ + H^+^

Δ*G*_acid_ is calculated using ωB97XD/6-311+G(3df,2p) model only, according to the following equation:

Δ*G*_acid_ = *G*^298^(A^−^) + *G*^298^(H^+^) − *G*^298^(AH)

The value for *G*^298^(H^+^), −6.28 kcal/mol, comes from the Sackur−Tetrode equation. It is important to note that stronger acids have lower ΔH_acid_ values, indicating that less energy is needed to release the acidic proton.

The standard energy difference method was used to calculate the energy of the hydrogen-bonding interaction between donors and acceptors

∆*H*_HB_ = *H*_AD_ − *H*_A_ − *H*_D_

where ∆*H*_HB_ is the enthalpy of bonding interaction, *H*_AD_ is the total energy of the H-bond complex, and *H*_D_ and *H*_A_ are the individual enthalpies of the donor and the acceptor, respectively. Since we are interested in estimating the energy of interaction in enzyme active site, the calculations of ∆*H*_HB_ are performed in diethyl ether using SMD-ωB97XD/6-311+G(3df,2p) model. Diethyl ether was chosen since it has a dielectric constant of 4.33, which resembles average dielectric constant in enzyme active site.

Similarly, the energy of π-π stacking is calculated as follows

∆*H*_π-π_ = *H*_π-π_ − *H*_C_ − *H*_benzene_

where ∆*H*_π-π_ is the enthalpy of π-π interaction, *H*_π-π_ is the total energy of the π-π complex between studied molecule and benzene, and *H*_C_ and *H*_benzene_ are the individual enthalpies of the studied molecules and the benzene, respectively. Again, SMD-ωB97XD/6-311+G(3df,2p) model diethyl ether was used. All calculations were performed using Gaussian 16 software [46].

## 4. Conclusions

In the previous research, resveratrol analogues **2**–**6**, as the molecules in focus, have shown the very strong inhibition potential of cholinesterases. In this research, experimental and DFT approaches for their p*K*_a_ value determination was carried out, knowing that it is very important for predicting their ADMET properties and their behavior in the environment. An in silico study was used to calculate more indicators about the absorption and distribution in the human body. The experimental p*K*_a_ values of the investigated resveratrol analogues range from 9.17 to 10.07. The obtained p*K*_a_ values are close, which is the result of the similarity of the chemical structures of the analyzed compounds. The applied UV/vis spectrometric method for the determination of the p*K*_a_ values showed satisfactory precision since the scattering values were less than 0.06 in all cases. Three computational models were used to calculate the p*K*_a_ values of resveratrol analogues, but only the ωB97XD/6-311+G(3df,2p) model produced satisfactory agreement with the experimental results, with a mean absolute deviation (MAD) of 0.4. However, while this model accurately predicted the least acidic species, the overall order of acidity did not fully align with the experimental data. This discrepancy is likely due to the small variations in p*K*_a_ values between the compounds, which result from a subtle interplay of mesomeric and inductive effects—factors that seem to exceed the accuracy of the models used. We identified that the errors may originate from inaccuracies in calculating inherent (gas-phase) acidity, suggesting that a more sophisticated computational approach is necessary to address this limitation.

## Figures and Tables

**Table 1:** Calculated values for the p*K*_a_ with the MarvinSketch 24.1.0. software for resveratrol analogues **2**–**6**.

Compound	Calculated p*K*_a_
**2**	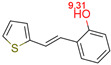
**3**	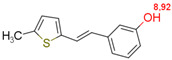
**4**	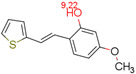
**5**	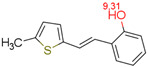
**6**	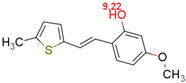

**Table 2:** In silico study for more indicators about the absorption and distribution in the human body of resveratrol analogues **2**–**6**.

Property	Model Name	Compound 2	Compound 3	Compound 4	Compound 5	Compound 6	Unit
Absorption	Water solubility	−3.45	−3.45	−3.46	−3.66	−3.69	log mol/L
Absorption	Caco2 permeability	1.67	1.67	1.78	1.67	1.79	log Papp in 10^−6^ cm/s
Absorption	Intestinal absorption (human)	90.34	90.34	90.23	91.01	90.91	% absorbed
Absorption	Skin permeability	−2.32	−2.32	−2.43	−2.26	−2.39	log Kp
Absorption	P-glycoprotein substrate	Yes	Yes	No	Yes	No	
Absorption	P-glycoprotein I inhibitor	No	No	No	No	No	
Absorption	P-glycoprotein II inhibitor	No	No	No	No	No	
Distribution	VDss (human)	0.52	0.52	0.41	0.59	0.48	log L/kg
Distribution	Fraction unbound (human)	0.08	0.08	0.08	0.07	0.07	Fu
Distribution	BBB permeability	0.60	0.60	0.59	0.59	0.57	log BB
Distribution	CNS permeability	−1.55	−1.55	−1.60	−1.55	−1.59	log PS

**Table 3:** Ionization constants of new resveratrol analogues **2**–**6**.

Compound	p*K*_a_	*R* ^2^	$\bar{{pK}_{a}}$ ± Scatter Value
**2**	9.85	0.99203	9.88 ± 0.04
	9.86	0.98737	
	9.92	0.98910	
**3**	10.10	0.96469	10.07 ± 0.03
	10.07	0.97490	
	10.05	0.95865	
**4**	9.11	0.99449	9.17 ± 0.06
	9.19	0.99321	
	9.21	0.98008	
**5**	9.45	0.99651	9.40 ± 0.05
	9.40	0.98647	
	9.36	0.96787	
**6**	9.62	0.95217	9.62 ± 0.04
	9.59	0.95614	
	9.66	0.90128	

**Table 4:** Calculated p*K*_a_ and ΔG_acid_ values for compounds **2**–**6**.

		Compound					
	Model	2	3	4	5	6	MAE
p*K*_a_	M1	8.6	9.7	8.1	7.9	8.1	1.1
	M2	8.7	10.2	8.9	9.3	8.6	0.4
	M3	8.4	9.9	9.0	8.3	7.5	1.0
Δ*G*_acid_ [kcal mol^−1^]	MII	337.8	346.7	338.0	338.8	337.0	

**Table 5:** Calculated enthalpies of π-π stacking and H-bond interactions for compounds **2**–**6** (in kcal mol^−1^).

		Compound				
	Model	2	3	4	5	6
∆*H*_π-π_ [kcal mol^−1^]	M2	−4.0	−3.9	−4.7	−4.0	−4.6
∆*H*_HB_ [kcal mol^−1^]	M2	−6.0	−4.8	−6.0	−5.9	−6.2

## Data Availability

The data are available from the authors on request.

## Supplementary Materials

The following supporting information can be downloaded at https://www.mdpi.com/article/10.3390/molecules29225481/s1. UV/Vis spectra of compounds **4**–**6**, the corresponding spectral difference, absorbance difference plots, a graphical presentation of species distribution as a function of pH (Figures S1–S3), the optimized geometries of all studied compounds **2**–**6** and Table S1: Bond distances (in Å) of neutral and deprotonated resveratrols 2 and 3 obtained by model M(II).
