# Supplementary material for: Experimental and DFT Approaches to Physico-Chemical Properties of Bioactive Resveratrol Analogues
Source: Molecules. 2024 Nov 20;29(22):5481. doi: 10.3390/molecules29225481 (PMC11597387; doi:10.3390/molecules29225481)
Supplement: Supplementary file 1 [file molecules-29-05481-s001.zip › molecules-3285029-supplementary.pdf]

Supporting Information

Experimental and DFT Approach to Physico-Chemical Properties of Bioactive Resveratrol Analogues

Borislav Kovačević,[1] Ivana Šagud,[2] Katarina Marija Drmić,[3] Milena Mlakić,[4] Irena Škorić[4,*] and Sandra Babić[3,*]

[1] Group for Computational Life Sciences, Division of Physical Chemistry, Ruđer Bošković Institute, Bijenička cesta 54, HR-10 000 Zagreb, Croatia

[2] Croatian Agency for Medicinal Products and Medical Devices, Ksaverska Cesta 4, HR-10 000 Zagreb, Croatia

[3] Department of Analytical Chemistry, Faculty of Chemical Engineering and Technology, University of Zagreb, Marulićev trg 19, HR-10 000 Zagreb, Croatia

[4] Department of Organic Chemistry, Faculty of Chemical Engineering and Technology, University of Zagreb, Marulićev trg 19, HR-10 000 Zagreb, Croatia

*Correspondence: Prof Irena Škorić; iskoric@fkit.unizg.hr; Prof Sandra Babić; sbabic@fkit.unizg.hr

Table of contents:

1. Spectral data and pK_a determination for compounds **4 – 6**
2. Optimized geometries of all studied compounds **2 – 6**
3. Table S1: Bond distances (in Å) of neutral and deprotonated resveratrols **2** and **3** obtained by model M(II)

1. Spectral data and pK_a determination for compounds 4 – 6

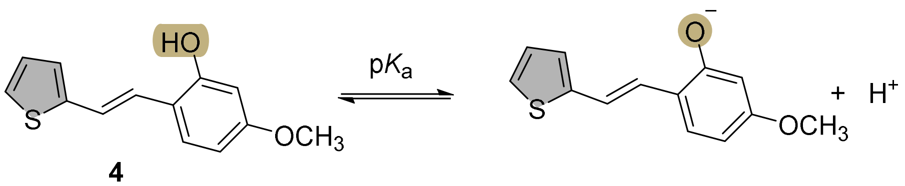

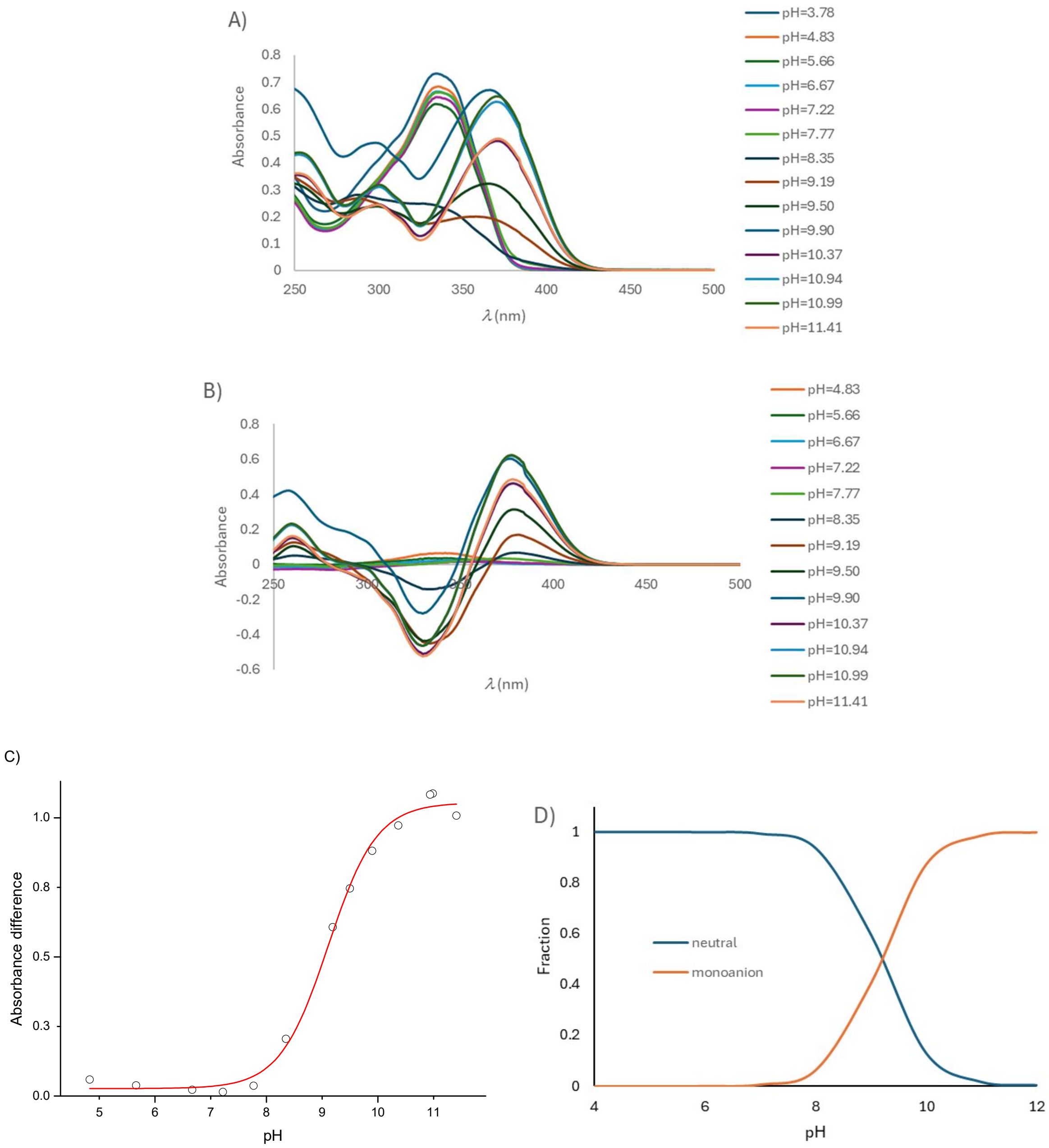

Figure S1. Spectral data and pK_a determination for Substance 4: (A) corrected spectra (A=0 for λ=500 nm), (B) spectral difference plot (maximum negative deviation at 330 nm and maximum positive deviation at 378 nm), (C) total absorbance difference *vs* pH, and (D) distribution of species as a function of pH.

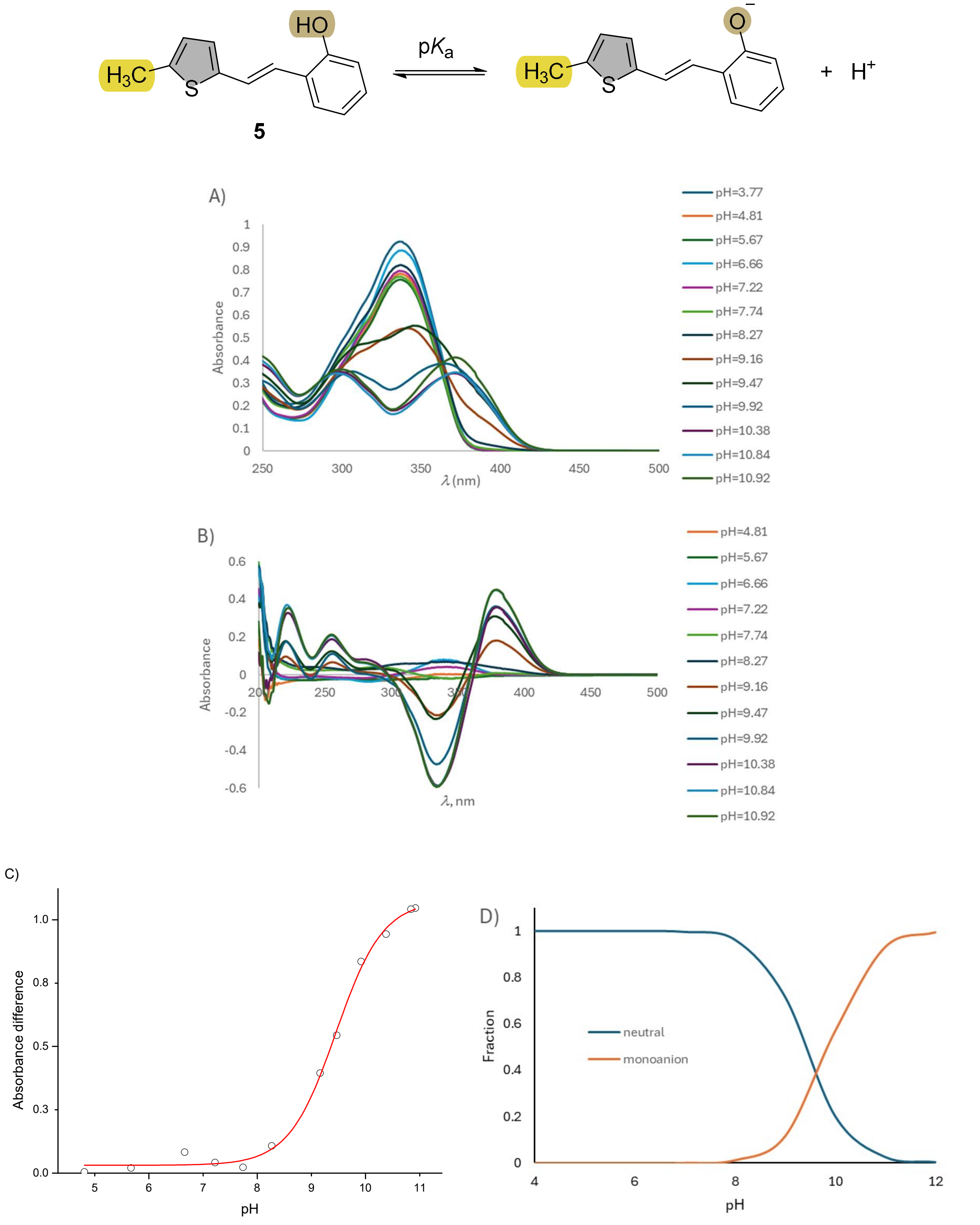

Figure S2. Spectral data and pK_a determination for Substance 5: (A) corrected spectra (A=0 for λ=500 nm), (B) spectral difference plot (maximum negative deviation at 332 nm and maximum positive deviation at 377 nm), (C) total absorbance difference *vs* pH, and (D) distribution of species as a function of pH.

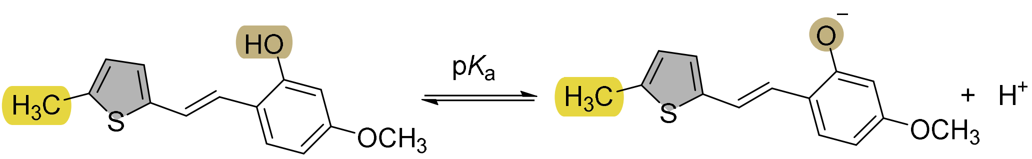

6

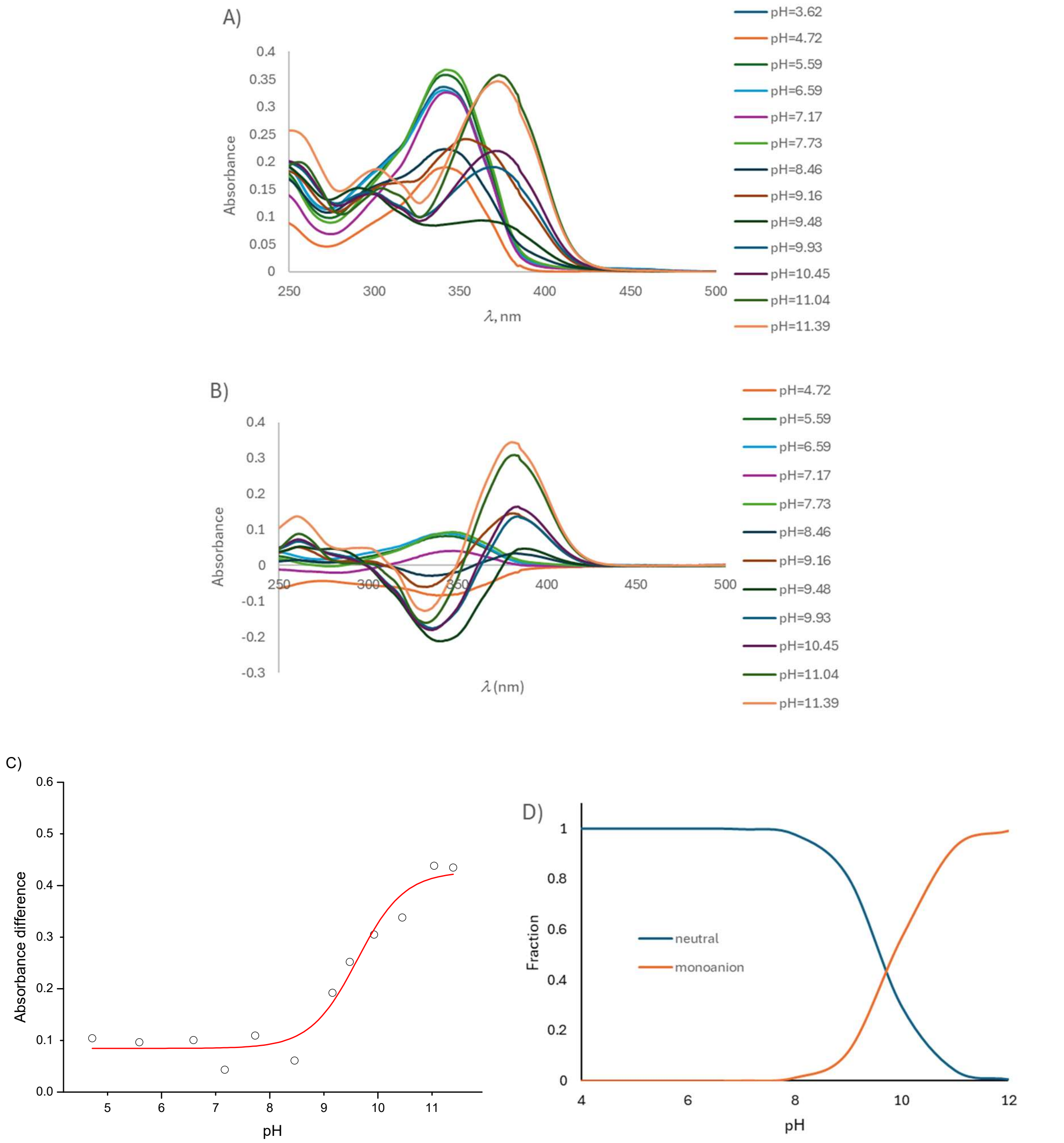

Figure S3. Spectral data and pK_a determination for Substance 6: (A) corrected spectra (A=0 for λ=500 nm), (B) spectral difference plot (maximum negative deviation at 339 nm and maximum positive deviation at 380 nm), (C) total absorbance difference *vs* pH, and (D) distribution of species as a function of pH.

2. Optimized geometries of all studied compounds 2 – 6

Geometries of molecules 2-6 optimized in water solution utilizing SMD-ωB97XD/6-311+G(3df,2p) model.

2-deprot-H2O

C -0.0233186709 -0.0669918646 0.0576296004

C -0.0100204709 -0.0281264364 1.4592062753

C 1.249052256 0.00755312 2.1432795794

C 2.4146786996 0.0017234391 1.3326259056

C 2.3586940973 -0.0372614341 -0.0448093881

C 1.129984987 -0.072447452 -0.7036134842

C -1.3032216043 -0.0268604784 2.1299854638

C -1.5897614655 0.0045016899 3.4415404118

C -2.9297315302 0.0027854187 4.0024544019

C -3.2452400572 0.034174088 5.3350625947

C -4.6416619897 0.0238949992 5.5882937307

C -5.3777178835 -0.0152498604 4.4463609852

S -4.3771305177 -0.0398009958 3.0552210535

O 1.3380166758 0.0435756841 3.4348728042

H -0.9870198336 -0.0937802974 -0.4405933219

H 3.375191617 0.0295490118 1.8345354353

H 3.2800365281 -0.0402238111 -0.6158452669

H 1.0781647057 -0.1031481385 -1.7837578995

H -2.141829093 -0.0561751713 1.4359875258

H -0.7922878062 0.0338746998 4.1698141446

H -2.4888729472 0.0638886663 6.1071615013

H -6.4509556202 -0.0310625486 4.3413561241

H -5.0777907295 0.0447959975 6.5767516212

H 2.8423309804 0.0638273073 4.1404766398

O 3.7014088164 0.0807651591 4.6391255967

H 4.3733601697 -0.0979950649 3.9791493502

2-H2O

S	0.0024429842	0.0172846893	-0.0051002936
C	0.0063004535	-0.0007764758	1.7234126602
C	1.2935541815	-0.0069033717	2.1903802271
C	2.268525224	0.0029760919	1.1604111311
C	1.7127817785	0.0164371436	-0.0804069671
C	-1.1929893763	-0.0079284087	2.5423090985
C	-2.4467113064	-0.0015735602	2.0699202833
C	-3.7172350081	-0.0065977038	2.7936758039
C	-4.8907214122	0.0045311215	2.0288239386
C	-6.150012177	0.0019610746	2.5993231448
C	-6.2639672141	-0.0119680618	3.9830496846
C	-5.1272289448	-0.023385613	4.7719706207
C	-3.8599140714	-0.0212918473	4.1936424002
O	-2.7500202243	-0.0332256224	4.9656189889
O	-3.2064727361	-0.0574754949	7.657405226
H	-4.794718704	0.0156404674	0.9495457568
H	-5.2088427949	-0.0336988223	5.8516261529
H	-7.2389536005	-0.0139249581	4.4530300386
H	-7.0317354386	0.0108474251	1.9731083263
H	-2.5813368064	0.009592606	0.9909432943
H	-1.0030155235	-0.0189982746	3.605829294
H	1.5254833839	-0.0181499416	3.2463437076
H	2.2114306547	0.0259369772	-1.0367145323
H	3.3347076657	0.0001498663	1.335064216
H	-2.9850537857	-0.0428226649	5.9177867781
H	-3.633468755	0.7785881286	7.8622386838
H	-3.8795410527	-0.7221988434	7.8262543865

3-deprot-H2O

C -0.000061382 0.0521390945 0.0028284093

C -0.0000150573 -0.0304501749 1.3948916899

C 1.2191639332 -0.1260753576 2.0738185099

C 2.4056512777 -0.1425768738 1.3509919976

C 2.4038513387 -0.0640810248 -0.029786957

C 1.1900644571 0.0379347066 -0.7550868373

C -1.2330388309 -0.0220925011 2.1934629802

C -2.4868483059 0.0096624619 1.7302142095

C -3.6950487863 0.016422965 2.5314559274

S -3.7072703783 -0.0021971546 4.2630014414

C -5.4310435723 0.016740848 4.3368943375

C -5.9591893731 0.0366201851 3.082545457

C -4.9768468196 0.0369809151 2.0577137978

C -6.1503208313 0.0130318612 5.6443338926

O 1.165540591 0.1153224377 -2.0587230719

O 3.4363998907 0.31579409 -3.3881314498

H -0.9337240936 0.13385751 -0.5419265391

H 1.231557619 -0.1887338506 3.1548162823

H 3.3392772929 -0.0787976682 -0.5773016458

H 3.3502617802 -0.2185613962 1.8771964475

H -1.070872725 -0.0496627783 3.2682173875

H -2.6741726975 0.0301838087 0.6613259077

H -5.2029052285 0.0510025872 1.0002427125

H -7.0248364204 0.0503150725 2.8996009162

H -5.9068942808 0.89515459 6.2389748573

H -7.2252793399 0.0077514278 5.4685727513

H -5.8981251258 -0.8659115747 6.2397982186

H 2.6052805898 0.2308006714 -2.8447837921

H 4.1382464781 0.0003501222 -2.8163408382

3-H2O

C -0.0005736463 0.0177903843 0.0091367113

C -0.0050225914 0.0073737135 1.3760015146

S 1.6205094986 0.0119421498 1.9716917464

C 2.2925513579 0.0272969294 0.3833794384

C 1.3027239949 0.0286870741 -0.5515786881

C -1.1790129366 -0.0048033607 2.2247413456

C -1.1862081889 -0.0212280293 3.5614869556

C -2.370659134 -0.031479945 4.4269932997

C -3.6728888482 0.0340772888 3.9287006888

C -4.759860835 0.019059196 4.7904435265

C -4.5660406006 -0.060293892 6.168918248

C -3.2738676152 -0.1226805156 6.6643342049

C -2.1827265373 -0.1080208202 5.8096014305

O -6.000863666 0.0853357994 4.2388179501

O -8.0232736232 0.1090762992 6.0887040466

H -3.8642562797 0.1021542859 2.8656579238

H -1.1778572423 -0.1570312117 6.2094592981

H -5.4179496921 -0.0715224757 6.8362918056

H -3.1180302855 -0.18345471 7.7338545126

H -0.24029034 -0.0319331199 4.0962709757

H -2.1133069547 -0.0018055001 1.6724233342

H -0.9121119845 0.0171987136 -0.5727346882

C 3.768892312 0.0395560043 0.1674194279

H 1.505004455 0.0372170754 -1.6136927883

H -6.6913487035 0.0845358529 4.933557872

H -7.8955410947 0.9248156583 6.5802886611

H -7.8119976076 -0.5875065683 6.7160564214

H 4.2297712344 0.9298801534 0.5984658856

H 4.2482280174 -0.8310420893 0.6178608255

H 3.9807481705 0.0301996807 -0.9009177153

4-deprot-H2O

C 0.0406006862 0.0128518517 -0.0028430888

C 0.0317836188 0.004800936 1.3669839786

S 1.6557121566 0.0028678646 1.9655279475

C 2.3192648893 0.0133496422 0.3846319301

C 1.3449895904 0.0180608353 -0.5628723386

C -1.14700243 -0.0023747694 2.2159173983

C -1.1272821634 0.0016891761 3.5591650386

C -2.2295931581 -0.0043153036 4.5086051496

C -3.6180536339 -0.0345852485 4.1387129043

C -4.5623931578 -0.0342309025 5.1898931144

C -4.1899928678 -0.0077276072 6.5228455908

C -2.8421585269 0.0202586089 6.8847971494

C -1.9040340422 0.0209723618 5.8662603267

O -4.0027373806 -0.0617016434 2.903646997

O -5.2133432649 -0.0105073446 7.4267006528

C -4.8871164565 0.0141237529 8.8053362721

O -6.5670583243 -0.3679519572 2.303621196

H 3.3888758991 0.0158765063 0.2462474534

H -5.616808907 -0.0532640685 4.9411679243

H -2.5249803538 0.0403111197 7.915918857

H -0.1512965448 0.0138568847 4.0425195475

H -2.0892574395 -0.0123635536 1.6864643191

H -0.8697748647 0.0149106837 -0.5862506252

H 1.5477319814 0.0244136913 -1.6242708362

H -5.6202741239 -0.2496113673 2.5808070996

H -5.8337039835 0.0067191373 9.339648964

H -4.3056187519 -0.8649961713 9.0907301581

H -4.3337879421 0.9188239319 9.0656073411

H -0.8539888599 0.0427271836 6.1379724251

H -7.0299672497 -0.6155773855 3.1058447152

4-H2O

C 0.0124117472 -0.0005286536 -0.0041336324

C 0.0082587963 -0.0002006626 1.3652557235

S 1.6308311079 0.0036574557 1.9623447551

C 2.2899350026 0.0048303352 0.3814030812

C 1.3150987135 0.002318678 -0.5660280679

C -1.1716241442 -0.0025230356 2.2126042909

C -1.1540300736 -0.001305596 3.5526828008

C -2.2610615903 -0.0029804399 4.5050055862

C -3.6327805662 -0.0023255878 4.1748468468

C -4.6004353009 -0.0033782605 5.1681948991

C -4.2391639294 -0.0051137147 6.5107363041

C -2.8956309221 -0.0057738112 6.8711214688

C -1.9450739122 -0.0046387005 5.8648105135

O -3.9883125761 -0.0009255594 2.8723748262

O -5.2663836299 -0.0061710195 7.3973565675

C -4.9530111579 -0.0079496029 8.7812207392

O -6.6778924615 0.0290695718 2.4189963867

H 3.3593585929 0.0071349262 0.2413852046

H -5.6492801525 -0.0029817871 4.900856746

H -2.5842762547 -0.0070136679 7.9042608087

H -0.1830868953 0.0011621752 4.0429798038

H -2.1061201961 -0.0052462937 1.670515617

H -0.8994636502 -0.0027241751 -0.5850774246

H 1.5169021632 0.0025816451 -1.6274415046

H -4.9642790052 0.0065492128 2.7726220013

H -5.9051587506 -0.008563005 9.3045875315

H -4.388162341 -0.9010098273 9.055113735

H -4.3880585206 0.8843492572 9.0573823674

H -0.8985455436 -0.0050033489 6.145207389

H -7.0587803193 -0.6401600272 2.9940469914

H -6.9949287724 0.863464336 2.7750407577

5-deprot-H2O

C 2.6385179816 0.2230599509 0.0451667415

C 1.7045941971 -0.8640106454 0.0214281553

C 2.1905535887 -2.1786870021 0.005114721

C 3.53950327 -2.4804810586 0.0110010776

C 4.4536034038 -1.4279487181 0.034286583

C 4.0158216657 -0.1198938231 0.0508504495

C 0.2570007762 -0.6946828452 0.0128238263

C -0.4716108493 0.4335519035 0.0245158764

C -1.9217393462 0.4946056049 0.0142339926

S -2.9532707611 -0.8983521893 -0.0154824351

C -4.3925944681 0.0558902857 -0.0118224584

C -4.0805699809 1.3796802911 0.0113688643

C -2.6821556178 1.6304771311 0.0261240281

C -5.7435147969 -0.5781387878 -0.0324457736

O 2.2613209161 1.4623658392 0.0613899664

H 1.4670155454 -2.9873043984 -0.0128755605

H 4.734921868 0.6911359526 0.0688927328

H 5.5177768736 -1.6339315461 0.0395062599

H 3.8750133836 -3.5088760334 -0.0020979693

H -0.2831843543 -1.6400907497 -0.0059059193

H 0.0232108732 1.3941728458 0.0431263698

H -2.2440539532 2.6190852817 0.0449993254

H -4.8342185313 2.15536683 0.0176778555

H 3.4163545783 2.6564051437 0.0787459427

H -5.9010163142 -1.2196326809 0.8362995386

H -6.5093991327 0.1965293345 -0.0252982404

H -5.8878357384 -1.1903419221 -0.9243175526

O 4.0404404573 3.4296474113 0.0838048206

H 4.911445466 3.043335594 0.189471782

5-H2O

C 0.0103290352 -0.0138247972 -0.0052070721

C 0.0064665798 -0.0059263956 1.3615951584

S 1.6338893206 0.0095600812 1.954267981

C 2.3044647278 0.0054122264 0.3644520623

C 1.3132730435 -0.0076030567 -0.5683577433

C -1.1708439776 -0.009306165 2.2090376958

C -1.1586360723 -0.0031814578 3.5492867502

C -2.276832676 -0.004817181 4.4919695976

C -3.6404009896 -0.0085177054 4.1433074641

C -4.6205417296 -0.0087051534 5.133290089

C -4.2724354768 -0.005123033 6.4726585846

C -2.9342897751 -0.0012315749 6.842740665

C -1.9643771113 -0.0011223739 5.8573913239

O -3.9818508769 -0.0119098524 2.8345581815

C 3.7805580427 0.0165146925 0.1461477136

O -6.6648902187 0.0264581383 2.333166242

H -0.9181555765 0.0021777895 6.1396721849

H -5.6620991209 -0.0117032735 4.8372467395

H -5.0500774935 -0.0053403229 7.225491189

H -2.6505245908 0.0017435987 7.8863794029

H -0.1918567154 0.0042648461 4.0472693827

H -2.1051814354 -0.0175713808 1.6662861244

H -0.9017917724 -0.0240186996 -0.5861281746

H 1.51360393 -0.0125043159 -1.6309559133

H -4.95643432 -0.0041088428 2.7252918918

H 4.2604985348 -0.8531694204 0.5978841991

H 3.9905210719 0.0039043653 -0.9226011137

H 4.2431859608 0.9076850833 0.5735731434

H -6.9852956425 0.8670190107 2.6710633677

H -7.0739525938 -0.6347685157 2.8978475503

6-deprot-H2O

C 0.0322347929 -0.0333500195 -0.0088858172

C 0.0114811944 -0.0146973137 1.3578920669

S 1.635944514 0.0075177203 1.9652562663

C 2.3252018694 -0.0084935196 0.3808788585

C 1.3429081891 -0.0297560011 -0.5592530791

C -1.170679811 -0.0131821937 2.2007634609

C -1.1602740255 0.0054276094 3.5447635714

C -2.2650955806 0.0089804314 4.4906242876

C -3.6479582126 -0.0081408555 4.1208580395

C -4.6085915765 -0.001757166 5.1692660875

C -4.2352889108 0.0198659532 6.4980010624

C -2.8833683502 0.0365442529 6.8594092485

C -1.9412190706 0.0307596509 5.8550251764

O -4.0353321823 -0.0285260585 2.8872687953

O -5.122901506 0.0265561228 7.5342189815

C -6.506377048 0.0114513246 7.2311484842

C 3.803959514 0.0009141808 0.1786577106

H -0.8915179923 0.04366948 6.128908012

H -5.6512496593 -0.0151096521 4.8847164898

H -2.6014300557 0.0534844839 7.903299877

H -0.1863122634 0.0204842096 4.0320926032

H -2.1083282468 -0.0286676168 1.663858024

H -0.873043455 -0.0491715046 -0.6004238276

H 1.5533610803 -0.0425519351 -1.6201273561

H -5.6619343834 -0.0735676781 2.543352958

H 4.278779431 -0.8704635251 0.6330951562

H 4.0259017542 -0.0099389954 -0.8878726868

H 4.2648515139 0.8901589482 0.612319981

H -7.0253353115 0.0202768556 8.1861757053

H -6.7948425823 0.8932293439 6.654937625

H -6.7807486439 -0.8900039465 6.6790565884

O -6.6120184953 -0.1128649068 2.2569557202

H -7.1140296738 0.1602186902 3.0266902606

6-H20

C 0.0088373765 0.0056449664 0.0025646216

C 0.0019096408 0.0039097656 1.405271473

C 1.2546085719 0.0041360161 2.0395943145

C 2.4381329215 0.004588415 1.3009717416

C 2.393996219 0.0062489623 -0.0845821936

C 1.1650275663 0.0070224252 -0.7440187667

C -1.2958970908 0.0014490379 2.074309688

C -1.5923961633 -0.0032115205 3.3819003016

C -2.9361552342 -0.0069045397 3.9305767899

C -3.2643987384 -0.0121269762 5.2572826128

C -4.6645772472 -0.0153519566 5.4954665649

C -5.4067599583 -0.0126080507 4.3550931024

S -4.3777137783 -0.0060229858 2.9692410432

C -6.8926312506 -0.0147203724 4.2163683503

O 1.3045160311 0.003924302 3.3891833405

O 3.4988116798 0.0072208963 -0.8717942762

C 4.771206323 0.0076002857 -0.2451536823

O 3.8194979714 0.0088414501 4.4515125695

H -0.9447936285 0.0059135447 -0.5117517726

H 3.3782940478 0.0031392654 1.8330897988

H 1.1357990322 0.0085017631 -1.8249329195

H -2.1258112359 0.002899042 1.3713625946

H -0.8100702427 -0.0049984069 4.1274045549

H -2.5162777514 -0.0136653535 6.0382246078

H -5.1110610211 -0.019611823 6.4803866507

H 2.2320111098 0.0065927547 3.7088550493

H -7.2448170862 -0.8939431578 3.6742401504

H -7.3507396787 -0.0189648055 5.2046398422

H -7.2479148717 0.8669965409 3.6803508454

H 5.5022914797 0.0090840575 -1.0488492841

H 4.906320065 0.8996743529 0.3697591228

H 4.9078312536 -0.8855558395 0.367831244

H 4.218905609 -0.8253124077 4.1905468646

H 4.3247336216 0.6779583653 3.9818450337

phenol-deprot-H2O

C -0.0783597141 -0.0509776781 0.0857376585

O -0.0961017291 -0.0664716097 1.3910275496

C 1.1350536487 0.0409618191 -0.6376334167

C 1.1560251199 0.0572861642 -2.0224632752

C -0.0218181682 -0.0164758119 -2.7598243619

C -1.2276347804 -0.1072216069 -2.0718819303

C -1.262881952 -0.1247990454 -0.6869114484

H 2.0626600079 0.0997270228 -0.078735949

H 2.108318468 0.1291296684 -2.5358111205

H -0.0004858245 -0.0029607928 -3.8415573336

H -2.158829452 -0.1659478835 -2.6241811755

H -2.214193055 -0.1971125178 -0.1712981706

H -1.5589230035 -0.0915929022 2.142108483

O -2.4006634572 -0.0984524683 2.6753902464

H -3.1033508791 -0.1807641097 2.0285807841

phenol-H2O

C -0.0275377429 -0.006216589 0.0234133399

C -0.009419846 0.0018448471 1.415913938

C 1.2051452573 0.0062297815 2.095752333

C 2.3928755177 0.00223485 1.3818483408

C 2.3862711476 -0.0060972824 -0.0074576439

C 1.1703287312 -0.0101663327 -0.6768904682

O -1.149585839 0.0050796324 2.1566539843

O -3.4392222467 0.0131682502 0.6454634633

H 1.2057632254 0.0127428776 3.1781210295

H 3.3328241816 0.0056901776 1.9191534848

H 3.316068841 -0.009250198 -0.5600207432

H 1.1481887738 -0.016623939 -1.7593818739

H -0.9738840676 -0.0094189283 -0.5025589454

H -1.940545205 0.0064106504 1.5791436653

H -3.3006601235 -0.6581241639 -0.0281074496

H -3.421553854 0.8451678087 0.1650061769

Geometries of molecules 2-6 optimized in water solution utilizing SMD-M062X/6-311+G(3df,2p) model.

2-deprot-H2O

C -0.0234810534 -0.059687678 0.0612373072

C -0.0074948757 -0.0340620726 1.4639611519

C 1.2515739392 -0.013888664 2.1485924415

C 2.4182084245 -0.0203954404 1.3385926282

C 2.3609197894 -0.0451540375 -0.0404254847

C 1.1306123425 -0.0653559564 -0.7006146558

C -1.2960727167 -0.0294178932 2.1416270812

C -1.5779515733 -0.0033757485 3.4562067326

C -2.9205973691 -0.0006729029 4.0109210233

C -3.2447470581 0.0274217516 5.3424168377

C -4.6450173212 0.022767937 5.5852296723

C -5.3714901485 -0.0089030964 4.4358371416

S -4.3598054814 -0.0333817307 3.0502490342

O 1.3357157107 0.0089910974 3.4411080319

H -0.9890699591 -0.0751636903 -0.4333222122

H 3.3779783931 -0.0054400777 1.8421435751

H 3.2817520097 -0.0491029502 -0.611624665

H 1.0794526765 -0.0850612203 -1.7805464148

H -2.1396354893 -0.0494758039 1.4531605391

H -0.7843263934 0.0183676437 4.1882333386

H -2.4916641945 0.0507121909 6.117935079

H -6.4435062862 -0.0194013045 4.3199523334

H -5.0901944483 0.0420256097 6.5694673383

H 2.8178616099 0.1112419535 4.1009946155

O 3.6880506843 0.1930821772 4.5846294777

H 4.3472881023 -0.1508843664 3.9763094369

2-H2O

S -0.0152382869 0.0121806056 0.0018118742

C 0.0096625519 -0.0010905132 1.7303971623

C 1.3020961598 -0.004210091 2.185672242

C 2.2672151476 0.0042876529 1.1437142199

C 1.6961319463 0.013724379 -0.0912812096

C -1.1842210151 -0.0076405718 2.5574826451

C -2.4384265898 -0.0037903209 2.08132934

C -3.7118900718 -0.0086404441 2.798287186

C -4.8847081105 -0.0012596276 2.0305935999

C -6.145266436 -0.0043362329 2.6011820151

C -6.2603602406 -0.0149231021 3.9864110271

C -5.1226540451 -0.0224899036 4.7771382848

C -3.855408642 -0.0198234212 4.1984639849

O -2.7435099631 -0.0278747281 4.9707822841

O -3.2296762993 -0.0504068859 7.6518656663

H -4.7843792367 0.0071857357 0.9517945959

H -5.2009420395 -0.0302532546 5.8573155782

H -7.2354888326 -0.0172391976 4.4554790836

H -7.026703286 0.0015672989 1.9750380605

H -2.5669486563 0.0047060057 1.0013563837

H -0.9881019084 -0.0158601931 3.619743686

H 1.5418995408 -0.0122656612 3.2399309376

H 2.1820219651 0.0214385331 -1.054008341

H 3.3353037513 0.0034720332 1.3051511645

H -2.9821790779 -0.0366399231 5.9238404153

H -3.6574435885 0.7886563601 7.8550275698

H -3.9074973427 -0.7154486049 7.8149725938

3-deprot-H2O

S -0.0028517103 0.1112417305 -0.0234883315

C -0.0512403466 -0.0598671127 1.6999207381

C 1.2194360825 -0.1514392467 2.1977198865

C 2.2250004815 -0.0848813719 1.1946546803

C 1.7225755027 0.0576124508 -0.0632669248

C -1.2801861456 -0.0978406571 2.4677536279

C -2.5185934786 -0.0177127038 1.9664112177

C -3.7746187171 -0.0578860978 2.7260995827

C -4.9758664972 -0.0321438544 2.0081699439

C -6.1844517983 -0.0796907246 2.6945743445

C -6.2199813388 -0.14957893 4.0764176727

C -5.0241586988 -0.1710863941 4.8393706231

C -3.8116878899 -0.1218784267 4.1196679751

H -2.8927902915 -0.1343985458 4.6941170695

H -4.9546906349 0.0199402784 0.9271161064

O -5.0380421086 -0.2366889921 6.1452879029

H -7.1693136447 -0.1925110795 4.5975460756

H -7.1149321554 -0.0639443384 2.1394546777

H -2.6444577569 0.0814365919 0.89080895

H -1.1266008035 -0.2057198746 3.5368447671

H 1.4181937486 -0.2640127686 3.2547848744

C 2.4664151115 0.1624808659 -1.3535687856

H 3.2864873149 -0.1410336928 1.3929437511

H 2.1906151432 -0.6397125293 -2.0391556649

H 2.2642800106 1.1102222043 -1.8542499788

H 3.5359935831 0.0970789493 -1.1606637596

H -6.4246405567 0.1526706273 6.8334087147

O -7.2517041717 0.4326572503 7.3301787771

H -7.9854676944 0.1717251839 6.7678451658

3-H2O

C -0.0034138888 -0.0218478424 -0.0031983397

C -0.0157848621 -0.0064572789 1.3646660959

S 1.6048960834 0.0359198326 1.9741061766

C 2.2877822804 0.0322987823 0.388967887

C 1.3059937806 -0.0001867527 -0.555111332

C -1.191195345 -0.0199986271 2.2111763712

C -1.1910477948 -0.0080760767 3.5494394687

C -2.3725366418 -0.0177575557 4.4178371879

C -3.6772895827 0.0081594789 3.9189361512

C -4.7616021373 -0.0035963328 4.7844254937

C -4.564721447 -0.0403145543 6.1654917737

C -3.2699563524 -0.0636326997 6.6611590108

C -2.1800751742 -0.0518467694 5.8026075846

O -6.0078193277 0.0226958452 4.2364311692

O -8.0028664304 0.0738660123 6.1093094097

H -3.8728959273 0.0420640212 2.8549782202

H -1.172627628 -0.0699336036 6.1980906989

H -5.418162555 -0.0494174795 6.8313004599

H -3.1114444463 -0.0911632732 7.7314136481

H -0.2419976587 0.008012472 4.0789565422

H -2.1267881171 -0.0432095857 1.661299936

H -0.9125331191 -0.0476054429 -0.5883163904

C 3.7668087521 0.0637101514 0.1883036889

H 1.5197442776 -0.0082179726 -1.6149797985

H -6.693965722 0.031641462 4.9370616999

H -7.8801081453 0.9066817753 6.5780074226

H -7.7771622992 -0.6041249712 6.7556083627

H 4.2072347978 0.9652092365 0.616082679

H 4.2506216424 -0.7950133666 0.6552293588

H 3.9876536217 0.0455351364 -0.8776464663

4-deprot-H2O

C 0.0395034732 0.0212264026 -0.0076736304

C 0.0216390073 0.0050975086 1.3629441403

S 1.6404953706 -0.0071294152 1.9761267587

C 2.3162663365 0.0102658751 0.3986153338

C 1.349995906 0.0246943928 -0.5582725804

C -1.161253783 -0.0014912856 2.2061867084

C -1.1377836122 -0.0045623641 3.5512677243

C -2.2355715698 -0.0083474346 4.5048427206

C -3.6239008284 -0.030116184 4.1317232876

C -4.5704938349 -0.0258029917 5.1810350555

C -4.1977811906 -0.0042747227 6.5151472192

C -2.849781636 0.0142238017 6.8815178819

C -1.9093179806 0.0116185207 5.8630598862

O -4.0015364469 -0.0529228968 2.8942856448

O -5.2220696809 -0.0019374679 7.4208356898

C -4.8812278699 0.019607497 8.7972520481

O -6.5464673418 -0.3681715644 2.3457762224

H 3.3870857434 0.0093169463 0.2710331689

H -5.6253457811 -0.0360589198 4.9335706524

H -2.5376398702 0.0299042779 7.9139048537

H -0.1591136751 0.0002859111 4.0295836903

H -2.1001209518 -0.0037420131 1.6717827007

H -0.8681859905 0.0306106517 -0.5951511796

H 1.5624742711 0.0367438021 -1.6175154202

H -5.5906611645 -0.245402735 2.6083173295

H -5.8209134046 0.0177310816 9.342413554

H -4.3005679025 -0.8627581996 9.0709582376

H -4.3179714948 0.9200513265 9.0473855296

H -0.8587181987 0.0263884768 6.132324015

H -6.9923015048 -0.6576414325 3.146060319

4-H2O

C 0.0141495571 -0.0135509994 -0.0072927451

C 0.0014117189 -0.0013153483 1.3629162332

S 1.6187974734 0.0219413277 1.9741608833

C 2.2898768373 0.0150751676 0.396319296

C 1.3227432806 -0.004439392 -0.5602522352

C -1.1825583431 -0.0066620927 2.204632427

C -1.1616038347 0.0029613373 3.5464543125

C -2.2642987856 -0.001420767 4.5027849084

C -3.63640535 -0.0048922924 4.1709792547

C -4.6055286959 -0.0088893928 5.162526746

C -4.2438074528 -0.0089845214 6.5065561832

C -2.9000532867 -0.0049554869 6.8698991436

C -1.9474536014 -0.0011473914 5.8632272557

O -3.9891677512 -0.004230921 2.8660711806

O -5.271551684 -0.0132198161 7.395340586

C -4.9429383797 -0.0138434415 8.7769422781

O -6.674415146 0.0473177049 2.4362765529

H 3.3605012032 0.025391137 0.267042729

H -5.6545775945 -0.0120929044 4.8943423448

H -2.5929808248 -0.0045698305 7.9041634066

H -0.1881040134 0.014579719 4.0320937091

H -2.1140696428 -0.0198336239 1.6580709994

H -0.895224527 -0.0287795148 -0.5919761056

H 1.5339285598 -0.0116493869 -1.6196330895

H -4.9670987809 0.0073360349 2.7670092728

H -5.8880028375 -0.0175250751 9.3117545843

H -4.3704459104 -0.9044750478 9.0396678382

H -4.3761347404 0.879778819 9.0418417954

H -0.9002838178 0.0021741933 6.1408922162

H -7.0648655211 -0.6252071665 3.0052926695

H -6.982582651 0.8837297891 2.8021684824

5-deprot-H2O

C 2.6393991183 0.2306463213 0.0923912859

C 1.7012234524 -0.8512893348 0.0337904582

C 2.1794708555 -2.1690853069 -0.0110577836

C 3.527945989 -2.477376455 -0.0020438023

C 4.4474368749 -1.4285962974 0.0564865122

C 4.0157474472 -0.1177991447 0.1028024533

C 0.2563823162 -0.6685877082 0.0190195341

C -0.4676064867 0.464756455 0.044006448

C -1.9180372839 0.517725688 0.0267158514

S -2.9338708822 -0.8874742883 -0.0221760343

C -4.3833337633 0.0535429648 -0.0148576199

C -4.0872592744 1.3815407573 0.0235570208

C -2.6894262383 1.6474698353 0.0471485674

C -5.7258392669 -0.5994299725 -0.048439641

O 2.2630404828 1.4700797629 0.1357407571

H 1.4495492005 -2.9706631126 -0.0548125203

H 4.7365373912 0.6903222626 0.1503231444

H 5.5101648666 -1.6400345661 0.0666376247

H 3.8594663311 -3.5060078638 -0.0381098069

H -0.2924355457 -1.6086225579 -0.0190140458

H 0.0219843498 1.427045741 0.0796583166

H -2.2584434917 2.6388947879 0.0784929114

H -4.8516636686 2.1465511569 0.0350786376

H 3.4152564809 2.6020182482 -0.0103910941

H -5.8718090817 -1.2577576831 0.8089630316

H -6.5006989328 0.165418077 -0.0292737453

H -5.8564428103 -1.1980643683 -0.9509403944

O 4.0593178573 3.3579443869 -0.1244773062

H 4.8958747127 3.0297692147 0.2153642392

5-H2O

C 0.0072541888 -0.0510831076 -0.0035154482

C 0.0036325444 -0.0218798881 1.3641495616

S 1.629948819 0.0317087749 1.9599430696

C 2.3015438908 0.013808684 0.3695106103

C 1.3127880629 -0.0313101021 -0.5660798318

C -1.170596159 -0.0316470215 2.2160439367

C -1.1397514562 -0.00943601 3.5576931897

C -2.24044381 -0.014883798 4.5193515327

C -3.608843165 -0.0262871473 4.1892840319

C -4.5762324059 -0.0290198446 5.1918359008

C -4.2092701574 -0.019776676 6.5279849603

C -2.8647428799 -0.0073678686 6.8799276991

C -1.9073102219 -0.0049742909 5.880802033

O -3.9665260518 -0.0341359613 2.8828956777

C 3.7790419284 0.0483142341 0.1576238004

O -6.6586264663 0.0657408962 2.4618713656

H -0.8565760923 0.0052890404 6.1450766688

H -5.6208542002 -0.0389440513 4.906246416

H -4.9761216458 -0.022193297 7.2914181524

H -2.5672736614 0.0004586448 7.9193940687

H -0.1643701002 0.0157292993 4.0385509414

H -2.1074673183 -0.0600546881 1.6786662563

H -0.9058923568 -0.0860102317 -0.5818843781

H 1.5180919282 -0.0498407887 -1.6275627083

H -4.9442281253 -0.014773102 2.7894970854

H 4.2701512044 -0.802129877 0.6320594089

H 3.992136405 0.0168930497 -0.9096465065

H 4.2191986055 0.9571964847 0.5698788856

H -6.9495990259 0.9098506881 2.8240298163

H -7.0703380337 -0.5970992491 3.0270450018

6-deprot-H2O

C 0.0238780152 -0.0034050075 -0.0197519257

C -0.0027941264 -0.0019705142 1.3481921058

S 1.6185727968 -0.0037953061 1.9670500237

C 2.3157554098 -0.0070302326 0.3847507074

C 1.339659841 -0.0061109361 -0.5632557303

C -1.1870062504 0.0004895386 2.187897043

C -1.1689217852 0.0048094152 3.5338395513

C -2.2661645385 0.0082575149 4.4870074589

C -3.6494146043 0.0024942212 4.1183452241

C -4.6108656108 0.0105561506 5.1671944546

C -4.2330291822 0.0194641969 6.4959404627

C -2.8794104893 0.0228447609 6.8572539513

C -1.937563019 0.0178776864 5.8515058887

O -4.0337624972 -0.0090770501 2.8834461045

O -5.1209525542 0.0259685887 7.5343619698

C -6.503304955 0.028461854 7.2202059194

C 3.7969799269 -0.0114987615 0.1953860173

H -0.8865730859 0.021623843 6.1199042433

H -5.6535926812 0.0092269892 4.8826712005

H -2.6013659157 0.0300825498 7.902006941

H -0.190763231 0.0067614072 4.0131539969

H -2.1229550769 -0.0015125973 1.6488127101

H -0.8801983951 -0.0024182727 -0.6132867614

H 1.5597155412 -0.0075061879 -1.6222144067

H -5.626314887 -0.1625983882 2.5941479617

H 4.2544336315 -0.8958637864 0.6411134156

H 4.0255682507 -0.0075842372 -0.8693085683

H 4.2613388253 0.8650254388 0.6493121193

H -7.0306068498 0.0345401691 8.1699655827

H -6.7727005793 0.9178974767 6.6479952807

H -6.7781346027 -0.8646109698 6.6563914817

O -6.5863193188 -0.2835743711 2.3473347707

H -7.0764411862 0.2414711873 2.9855591369

6-H20

C 0.0014460171 -0.0023715627 0.0040625767

C -0.0001836212 -0.000274442 1.4080848899

C 1.2541101117 0.0028104005 2.0398794498

C 2.4375408823 0.0026285058 1.3001581837

C 2.3884744328 0.0005498996 -0.0867700472

C 1.1571883063 -0.0018293895 -0.7448036329

C -1.2926086187 -0.0017594525 2.0857839916

C -1.5828438878 -0.0025048869 3.3966204311

C -2.9281341723 -0.0051851796 3.9414652502

C -3.2621145498 -0.0064850539 5.2678411183

C -4.6656989199 -0.0094795953 5.4984439959

C -5.4001609201 -0.0104546998 4.3520706368

S -4.3638430465 -0.0077657868 2.9699239102

C -6.8854996417 -0.0134832179 4.2003560059

O 1.3048123004 0.0059725323 3.3910395921

O 3.493580667 0.0006793088 -0.8767005154

C 4.7626142422 0.0040269485 -0.240511775

O 3.8196063065 0.0213811325 4.4317430346

H -0.9550888915 -0.0044333641 -0.5046482943

H 3.3778504004 0.0037796286 1.8326018062

H 1.1308361835 -0.0033141104 -1.8257051349

H -2.1280869996 -0.0030843636 1.3891566075

H -0.802431294 -0.0014980664 4.1438025539

H -2.5153931836 -0.0053514078 6.0501349873

H -5.1210877828 -0.0108847859 6.4792557084

H 2.2351880622 0.0116492896 3.7083833251

H -7.2292970078 -0.894629877 3.6570882471

H -7.3497098543 -0.0146472351 5.1852694749

H -7.2329398789 0.8664495836 3.6574436928

H 5.5000832634 0.0042753238 -1.0375352

H 4.8871444444 0.8973995448 0.3735336513

H 4.8905592706 -0.8870515613 0.3761364294

H 4.225817978 -0.8162544058 4.1832961

H 4.3289849449 0.6894883592 3.9598889268

phenol-deprot-H2O

C 0.0174475811 -0.1642656129 -0.0018615125

C 0.0183206137 -0.1273035697 1.4139945126

C 1.2826664273 0.0022270284 2.0374212141

C 2.4516996609 0.0880510453 1.2981175384

C 2.4262616282 0.0505120238 -0.0937911746

C 1.1942261729 -0.0764334613 -0.7296598372

O -1.0824982005 -0.209185084 2.1142997885

O -3.3513794921 -0.1226561544 0.8592739449

H 1.3170772462 0.0333652415 3.1205211805

H 3.3986888985 0.1862200277 1.816094486

H 3.340945663 0.1184338586 -0.6667765135

H 1.1488725426 -0.1086933183 -1.8121031019

H -0.9319691126 -0.2654926159 -0.5154620685

H -2.4478962921 -0.1698100253 1.2986148856

H -3.214998481 -0.4490867753 -0.0339040747

phenol-H2O

C -0.0282180034 -0.0094618925 0.0221519824

C -0.0105347276 -0.0004826252 1.4150636388

C 1.2030584338 0.0060594895 2.0974195467

C 2.3926975105 0.0037535778 1.3847244391

C 2.3870272142 -0.0052927613 -0.0058189019

C 1.1712120348 -0.0118742545 -0.6777978464

O -1.1546640469 0.0015810854 2.1536121907

O -3.4169539154 0.0179614512 0.6134519725

H 1.1981019177 0.0130691671 3.1797018629

H 3.3318650125 0.0090535476 1.9228140336

H 3.3172235186 -0.0070502455 -0.5570794653

H 1.1505747866 -0.0190563358 -1.7600467326

H -0.9762410456 -0.0149453276 -0.5012006642

H -1.9430712554 0.0023900447 1.5702826744

H -3.2787094526 -0.6573785069 -0.0598716011

H -3.3858303948 0.8512750068 0.1307025845

Geometries of molecules 2-6 optimized in water solution utilizing SMD-B3LYP-D3/6-311+G(3df,2p) model.

2-deprot-H2O

C -0.0124514528 -0.0024757687 -0.0001055172

C -0.0030268205 -0.0001261995 1.4417680509

C 1.2363842983 0.0015651866 2.1111330701

C 2.4510846751 0.0008938538 1.4466196223

C 2.4470190376 -0.0019439869 0.0479960677

C 1.2528852194 -0.0038750427 -0.6494602622

C -1.196895354 0.0009183167 2.265846349

C -2.5002537872 0.0003405777 1.9068634865

C -3.624737774 0.001175996 2.8144185137

S -3.4903553375 0.0030745396 4.5576310263

C -5.2050857563 0.0028620416 4.7602074495

C -5.8489899301 0.0013925782 3.5576658486

C -4.9551358799 0.0004687462 2.4554307216

O -1.115795142 -0.003114342 -0.6886650188

O -1.0710171373 0.0366492509 -3.3392417844

H 1.2267028923 0.0035552026 3.195768343

H 1.2579500142 -0.0068729904 -1.7326907753

H 3.383833028 -0.0028824683 -0.4967185339

H 3.381112087 0.0024420113 1.998990123

H -0.9769803864 0.0023985688 3.3314980657

H -2.7674284135 -0.0008433021 0.8609146192

H -5.270234998 -0.0008239088 1.4214835909

H -5.6313991006 0.0037944087 5.7505329773

H -6.9252433612 0.0009163983 3.4615865402

H -1.0327634865 0.0229630795 -2.339631885

H -0.1638882346 0.1955940404 -3.6184934956

2-H2O

S 0.0015889951 -0.0036391406 0.002725274

C	-0.0002949177	-0.0009769863	1.7488242534
C	1.2958913982	-0.0017445599	2.2144695795
C	2.2737377166	-0.0045230105	1.1873098936
C	1.7246286019	-0.0059314378	-0.0618771068
C	-1.1906401974	0.0013259652	2.5682984256
C	-2.4594412747	0.0019810459	2.1119032574
C	-3.7178109045	0.0029112633	2.8452343021
C	-4.9053786061	0.0013079111	2.0895580985
C	-6.1618908539	0.0009995218	2.6723347932
C	-6.266142447	0.0021134153	4.0608908002
C	-5.1188808035	0.0038391247	4.8417644005
C	-3.8537074331	0.0047024992	4.2530992788
O	-2.7316796117	0.0072549262	5.0225322237
O	-3.1831655848	0.0040315281	7.7253730086
H	-4.8197745563	0.0001358321	1.0098905068
H	-5.1895429041	0.0041512963	5.9215077761
H	-7.237031039	0.0015937929	4.5386222293
H	-7.0486200067	-0.0002688065	2.0533444941
H	-2.605010989	0.0009970301	1.0351605929
H	-0.9920051727	0.0022381216	3.6294159018
H	1.5268455274	-0.0003763191	3.2703278102
H	2.2279815723	-0.0080787492	-1.0154673546
H	3.3389543547	-0.0055073981	1.3671546467
H	-2.9611039153	0.0071049456	5.9802736774
H	-3.6133583377	-0.8372147042	7.9214902535
H	-3.8606721492	0.6695410084	7.8967626512

3-deprot-H2O

S	0.0108778413	0.0488567829	-0.0672677671

C -0.0631615456 -0.0081482156 1.679317236

C 1.2118421354 0.0058625021 2.1936564933

C 2.2287935552 0.0607017096 1.2052847816

C 1.7493885868 0.0898394978 -0.0737143765

C -1.2872942302 -0.0610570766 2.4403040238

C -2.5407806797 -0.0827334619 1.950931402

C -3.7856332272 -0.1361938331 2.7181207246

C -4.9980359268 -0.1821893562 2.0082694613

C -6.2026994104 -0.2376317017 2.7037859336

C -6.2316700673 -0.2469214484 4.0902975001

C -5.0294555801 -0.198392111 4.8478166434

C -3.820691742 -0.1429878633 4.1180766151

H -2.9010603915 -0.1043714216 4.6888105579

H -4.9883157295 -0.1764491652 0.9258715237

O -5.0344040091 -0.2059674315 6.1615763585

H -7.17666997 -0.2945993543 4.6173977045

H -7.1351875053 -0.2752756936 2.1527868535

H -2.6784852048 -0.0614023875 0.8734541338

H -1.1270350324 -0.0859016549 3.513096965

H 1.4028308687 -0.0234609365 3.2573585762

C 2.5171567123 0.1499773187 -1.3525425572

H 3.2865108032 0.0781733696 1.4280138949

H 2.278141761 1.0485573589 -1.9251645294

H 3.585335178 0.1583396473 -1.138382562

H 2.3051791574 -0.7092182382 -1.9921640842

H -6.4578751315 0.1359409183 6.8834404412

O -7.2952659989 0.3648513187 7.3888715009

H -7.9396146781 0.5868397195 6.7094002311

3-H2O

C	0.0060964154	0.0209286271	0.0117048069
C	0.0026975848	0.0039597523	1.386775359
S	1.6487711963	-0.0015484154	1.9764781021
C	2.3094952948	0.0190777905	0.3691067185
C	1.3049941205	0.0292066094	-0.5576649756
C	-1.1645557418	-0.005575436	2.2320471005
C	-1.184871298	-0.0244964263	3.5774546811
C	-2.3682555326	-0.0299639181	4.4342340725
C	-3.6764120429	0.0206709259	3.9319183239
C	-4.7675871049	0.0123823966	4.7913759923
C	-4.5797715259	-0.0457203954	6.1759922985
C	-3.2852108358	-0.0941195944	6.6779112261
C	-2.1885193926	-0.0860507543	5.8258257038
O	-6.0152957359	0.0635549256	4.2293491747
O	-8.0426147565	0.1054288849	6.0902931436
H	-3.8654801158	0.0707568019	2.8683871229
H	-1.1859592096	-0.1239945607	6.2315533583
H	-5.434726035	-0.0517811354	6.8380812092
H	-3.1330670154	-0.138481299	7.7486002743
H	-0.2417556828	-0.0391456428	4.1154592494
H	-2.0972623785	0.0032297449	1.6778544668
H	-0.9080359769	0.0273833157	-0.5653841334
C	3.7843028437	0.0255530141	0.1392845291
H	1.4980767417	0.0424987681	-1.6211489696
H	-6.7115899621	0.0706160455	4.9236448502
H	-7.9058848476	0.9269435134	6.5778413899
H	-7.8227091373	-0.5904085969	6.7217395638
H	4.2571580096	0.9052653672	0.5806725267
H	4.2643511782	-0.8539425793	0.5730406658
H	3.9883615769	0.0311662917	-0.9307936608

4-deprot-H2O

C -4.0951444182 1.1723530776 0.0237035561

C -3.1313408957 0.1878147008 -0.0081815637

S -3.9024693019 -1.3800535662 -0.0868303632

C -5.4829975435 -0.6829889913 -0.0749792589

C -5.4253734595 0.6783268343 -0.0139305386

C -1.7013309987 0.3934722871 0.0177231602

C -0.7658480834 -0.5831495345 -0.0107857434

C 0.679686421 -0.4920634827 0.0099541333

C 1.4200826944 0.7476875611 0.0629175425

C 2.83242742 0.6559207326 0.0763703548

C 3.5009660719 -0.5589962508 0.0412028028

C 2.7888466711 -1.7629146428 -0.0103020297

C 1.4036838375 -1.6937869112 -0.0244949386

O 0.8286105624 1.9043818554 0.0969734899

O 4.8739136288 -0.4862841846 0.0606358016

C 5.6190021223 -1.7039037497 0.0263306234

O 2.2292872411 4.1512988923 0.0880809417

H -6.353542674 -1.3179879782 -0.113332998

H 3.4106534446 1.5701200208 0.116151639

H 3.2854123825 -2.7195749849 -0.0387330102

H -1.1255471265 -1.6093624217 -0.0558658466

H -1.3953188082 1.4283378551 0.0633235129

H -3.841551304 2.2219362885 0.0729841192

H -6.3031514147 1.3082806154 0.0033480014

H 1.7502289293 3.272509485 0.0940513185

H 6.6662767804 -1.4137437686 0.0498245066

H 5.3973028322 -2.3283832575 0.8939168227

H 5.4195928709 -2.2644802593 -0.8890022483

H 0.845777551 -2.6226712082 -0.0645250897

H 3.1635967533 3.9232375715 0.1233128246

4-H2O

C 0.0551525763 0.0075797464 -0.0028819155

C 0.0353860208 0.0005750739 1.3743821414

S 1.6718445524 -0.0063233242 1.9851321616

C 2.3381433001 0.000240626 0.3935784372

C 1.3607196225 0.0074361199 -0.5580635267

C -1.1488682123 -0.0004077186 2.2030721097

C -1.1637175795 -0.0061912631 3.5522228208

C -2.2839831505 -0.005689306 4.4792766047

C -3.6518982377 -0.0022248134 4.1294284101

C -4.6496190479 0.0004148092 5.1071209804

C -4.3091097966 -0.0020577928 6.4550465122

C -2.963985108 -0.006702839 6.835850736

C -1.9917666916 -0.0082367461 5.8578825825

O -3.9926099313 -0.0021710315 2.8141832191

O -5.2232917028 -0.0004736109 7.4687337894

C -6.6137224532 0.0024155215 7.1353092847

O -6.6905293619 0.0145332252 2.3404756162

H 3.4075889076 -0.001629301 0.2569105974

H -5.680380911 0.0047280182 4.7888999951

H -2.705947783 -0.0089128476 7.8855719212

H -0.203199382 -0.0109131164 4.0610434885

H -2.0734504057 0.0047172015 1.6456108573

H -0.8522011547 0.0127706209 -0.5901208392

H 1.5661764256 0.0124500222 -1.6187007321

H -4.9711274858 0.0021295792 2.699120981

H -7.1464380007 0.0019592872 8.0821692628

H -6.8792290531 0.8963062098 6.5680131009

H -6.8822620892 -0.8889208455 6.5653947015

H -0.9510753789 -0.0114817357 6.1572912971

H -7.0777308581 -0.6582620822 2.9139568058

H -7.015561113 0.8516772855 2.6938574563

5-deprot-H2O

C 2.6476920031 0.2238351581 0.0541316099

C 1.7093304416 -0.8705814256 0.0262641958

C 2.206918884 -2.1878411606 0.0082777322

C 3.5602500564 -2.482628279 0.0161613877

C 4.4739197941 -1.424506211 0.0434232382

C 4.0285158078 -0.1150109852 0.0618501553

C 0.2669764716 -0.7089984378 0.015411301

C -0.4782890653 0.4192881546 0.0271347182

C -1.9206241287 0.4809636361 0.0149764185

S -2.9737132642 -0.9185827417 -0.0171034644

C -4.4142215011 0.0587605537 -0.0143368689

C -4.0846231248 1.3834888698 0.0100817779

C -2.6848923192 1.6244120858 0.0265934561

C -5.7716162551 -0.5627993729 -0.0366226955

O 2.2653583794 1.4674645155 0.0716989351

H 1.4888781354 -3.0006277483 -0.012657968

H 4.7418736724 0.6998725717 0.0831197273

H 5.5384803477 -1.6270107348 0.0503033054

H 3.90027595 -3.5094253158 0.0016272344

H -0.2678461062 -1.6564964971 -0.0053061414

H 0.0115333407 1.3814413193 0.0474534261

H -2.2419809919 2.6105042325 0.0465842039

H -4.8310123958 2.165847512 0.0160381489

H 3.4109939946 2.6590415626 0.0879687874

H -5.9378102616 -1.2048066457 0.8310769305

H -6.5319442137 0.2176843019 -0.0306229391

H -5.9221241995 -1.1760113389 -0.9276997052

O 4.0351635363 3.4411854964 0.096903377

H 4.916468012 3.0544739243 0.0978527155

5-H2O

C 0.0081232678 -0.0033845586 0.0070511208

C -0.0055615551 0.0005097277 1.4162291768

C 1.2542266306 0.0037000835 2.0529500356

C 2.440229898 0.004887166 1.3151309586

C 2.400014839 0.0009757045 -0.0745688401

C 1.1693116352 -0.0034978667 -0.7372536796

C -1.3005157371 0.0016771315 2.0785363569

C -1.609079531 0.0081949227 3.3925363612

C -2.9437971775 0.0107567875 3.9437453212

C -3.2680491257 0.0181316778 5.2798893449

C -4.665170556 0.0197099754 5.5330899685

C -5.4281923524 0.013554349 4.4004162337

S -4.4108510349 0.0056992035 2.9892678906

C -6.9163060532 0.0133429198 4.2804481053

O 1.3034251186 0.0057869467 3.4116106112

O 3.5123444292 0.0012007098 -0.866822793

C 4.7974832715 0.0045508072 -0.2404138285

O 3.8357657124 0.0080360211 4.4646356129

H -0.9430744716 -0.006381454 -0.5105072146

H 3.3772924576 0.0095429694 1.8498700323

H 1.1444374701 -0.0067260638 -1.8179617352

H -2.1275849068 -0.0024548295 1.3734975716

H -0.8277301786 0.0126548456 4.1379369545

H -2.5134227144 0.0223643006 6.0540733255

H -5.0988696736 0.0252501273 6.5234153091

H 2.2345199689 0.0053937058 3.7322447943

H -7.2790650247 -0.8683071467 3.7479125406

H -7.3640964574 0.0175604876 5.2736715645

H -7.2786511414 0.8907676208 3.7406761649

H 5.5226229479 0.0031497989 -1.0494512289

H 4.9340796991 0.8989387583 0.3702047624

H 4.9362392467 -0.8860522723 0.3752534239

H 4.3353409466 -0.6644329521 3.9854103906

H 4.2299408389 0.8452016878 4.1905376773

6-deptot-H2O

C 0.0358657433 -0.0399542744 -0.0062334281

C 0.0199320204 -0.0196874374 1.3691370606

S 1.6680175109 0.0065922056 1.9649276414

C 2.3410648196 -0.0102905303 0.3585952918

C 1.3406172504 -0.0346601875 -0.5695328851

C -1.1523007886 -0.0194609423 2.2121348196

C -1.1519737379 0.0011876297 3.5654832384

C -2.2569618646 0.0036633469 4.5020418422

C -3.6468463593 -0.0168418969 4.1260815884

C -4.6139568288 -0.0119056313 5.1711524936

C -4.2475788297 0.0129042006 6.5053310483

C -2.894622663 0.0336381608 6.8743247146

C -1.9427771505 0.0283299308 5.8750062054

O -4.0287831548 -0.0391347074 2.8847267644

O -5.1445765039 0.0191476472 7.5465101932

C -6.5389752906 -0.0072012085 7.2427371304

C 3.81792698 0.0023350051 0.1383550259

H -0.8957684006 0.0439529115 6.1566833156

H -5.6531836442 -0.0288943783 4.8798551908

H -2.6213828465 0.0529780396 7.9201263718

H -0.1807801315 0.0191858731 4.0563807964

H -2.089785959 -0.0375078821 1.6763366669

H -0.8738503549 -0.0583360517 -0.5902458955

H 1.5380738439 -0.0485176951 -1.6326635318

H -5.6456221334 -0.0111669748 2.530800625

H 4.3035701978 -0.8639651831 0.5928467133

H 4.0291721545 -0.0138594033 -0.930525285

H 4.2838651672 0.8946009812 0.5622527315

H -7.0539671743 0.0009043028 8.1999109626

H -6.834274837 0.8704616027 6.6642813171

H -6.8067338176 -0.9123100626 6.6939536161

O -6.5997974762 0.002993561 2.2318011265

H -7.1068589248 0.1801154183 3.030354865

6-H2O

C -0.0302328396 0.0353791151 0.0328757438

C -0.0205764411 0.0163688931 1.407647958

S 1.6342484614 -0.0023689979 1.9773784093

C 2.2787389639 0.015644354 0.3616201526

C 1.2636905649 0.0348461231 -0.5518704399

C -1.1813937138 0.0123010014 2.266658182

C -1.16094458 -0.0078972632 3.616118275

C -2.2553061462 -0.0137455512 4.5740760578

C -3.632323436 0.0045023858 4.2642421604

C -4.6025985658 -0.0032302345 5.268704739

C -4.2246873204 -0.0288984262 6.6065089508

C -2.8694840137 -0.0471978892 6.948675204
C -1.9248563631 -0.0394432385 5.9437987948
O -4.011479562 0.0297389209 2.9590224678
O -5.1094042363 -0.0379036071 7.6466723761
C -6.5088532452 -0.0190631903 7.3544526211
C 3.7514826473 0.0096760835 0.116920216
H -0.8762103131 -0.0535747566 6.2136074797
H -5.6413693711 0.0116139805 4.9781046014
H -2.5819226343 -0.0669902312 7.9905346212
H -0.1870736234 -0.0227636795 4.0983091629
H -2.1205367798 0.0270921221 1.7337791936
H -0.9501284362 0.0492607416 -0.5349667703
H 1.4441320449 0.0482970736 -1.6177653366
H -4.992215994 0.0468435222 2.8710116412
H 4.2278963285 -0.8781097107 0.5380701729
H 3.9449952126 0.0219378592 -0.9551915661
H 4.2386045308 0.8807927345 0.5600828728
H -7.0135576504 -0.0298546964 8.3165080576
H -6.783531474 0.8852804434 6.8085175341
H -6.8014268403 -0.8993089528 6.7791768743
O -6.7145897036 0.0922689569 2.5201704818
H -6.9699511258 -0.7727489606 2.1774707528
H -6.8523792403 0.7002890055 1.7838170776

phenol-deprot-H2O

C 0.0764557508 0.8708264882 0.0604261998
C 0.2329574183 -0.5402866177 0.0533772081
C -0.9621239069 -1.3036677656 -0.001644992
C -2.2113897329 -0.6995278461 -0.0468110486
C -2.3391368593 0.6900303131 -0.0397808464

C -1.1790149736 1.4629620838 0.014356039

O 1.4122559398 -1.1150040327 0.0955176279

O 3.5813439206 0.3764581688 -0.0154680955

H -0.8776823486 -2.3843538967 -0.0083018404

H -3.0986165482 -1.3208801738 -0.0886270967

H -3.3143777638 1.1567835449 -0.0760398645

H -1.2535429635 2.5443894621 0.0211647415

H 0.9657550206 1.4883535127 0.1035007801

H 2.7264726911 -0.1486513854 0.0353072993

H 3.3332629777 1.2754875921 0.221871689

phenol-H2O

C -0.048663271 -0.0054345365 0.0377623167

C -0.0191118044 0.0036863308 1.4337761402

C 1.2031914583 0.0066494587 2.105915608

C 2.3887896275 0.0003360952 1.3815685914

C 2.3711100697 -0.0089865288 -0.011276167

C 1.1467709112 -0.0117520572 -0.6734737489

O -1.1599606084 0.0094360793 2.1904805675

O -3.4648622591 0.0206440551 0.683782921

H 1.2099366691 0.0139200865 3.1878424733

H 3.3326481161 0.0027450095 1.9114647658

H 3.2964211662 -0.0138751588 -0.5709286265

H 1.1166316028 -0.018956026 -1.7555471476

H -0.9995610243 -0.0074268735 -0.4783247068

H -1.9599602381 0.0117047709 1.6188435122

H -3.3227377738 -0.6517678005 0.0061998531

H -3.4448838411 0.8569078529 0.2025238335

Geometries of π-π complexes between molecules 2-6 and benzene, optimized in diethyl ether solution utilizing SMD-ωB97XD/6-311+G(3df,2p) model.

2+benzene

C -0.0707908711 -0.0129638046 0.060378126

C 0.0042231585 -0.0130220412 1.4456845251

C 1.2422832108 -0.0118881381 2.0746842639

C 2.405144443 -0.0131115112 1.3174902117

C 2.3300085089 -0.0149537546 -0.068141537

C 1.0928034333 -0.0133864912 -0.6960113296

C 1.0714217186 3.4105750439 -1.1551731236

C 1.1867357914 3.4661039194 0.243257592

C -0.0042219824 3.505844065 0.9758034384

C -1.2495423201 3.4897969825 0.3766156978

C -1.3335862947 3.4297387993 -1.0061517432

C -0.1769818004 3.3909854868 -1.7648669966

C 2.4392181216 3.455430548 0.9942146911

C 3.700144351 3.3980799999 0.5505018473

C 4.8767494566 3.338038828 1.3979607576

S 4.8227584915 3.3038689727 3.1251678566

C 6.5282142521 3.2274658401 3.2484923338

C 7.1194017421 3.2250782799 2.0244657343

C 6.1757713448 3.2873324733 0.9680969361

O 2.2014476173 3.3739604231 -1.9021491112

H 0.0649300473 3.5391274537 2.0565096807

H -0.2363355266 3.342843285 -2.8467777382

H -2.2970292835 3.4125608628 -1.4992511245

H -2.1453465883 3.5180970436 0.9822373285

H 2.2815339903 3.471685921 2.0696682547

H 3.9091729938 3.3749194756 -0.5091545827

H 6.438868634 3.2966224182 -0.0807054865

H 6.9997915362 3.1880677333 4.2176641167

H 8.1893521765 3.1808768392 1.8803494885

H 1.9701819438 3.3383361827 -2.8334117792

H 1.3007567859 -0.0054867764 3.155977943

H 3.3711332442 -0.0005686513 1.8063838185

H 3.2374639835 -0.0031604759 -0.6583666384

H 1.0340290175 -0.0037116838 -1.7772088033

H -1.0355795076 -0.0001213674 -0.430332221

H -0.9032575454 -0.0040513107 2.036331976

3+benzene

S -0.0088931709 -0.0104288565 -0.0206709336

C -0.0086319435 -0.0123423266 1.7085871157

C 1.2756065759 -0.0262766476 2.1743703955

C 2.2506873859 -0.0321172047 1.144052691

C 1.7130747153 -0.0251731182 -0.1065022679

C -1.2086894174 0.0344855137 2.5167369536

C -2.4620807643 0.1239976096 2.0651610588

C -3.6722820859 0.2353798179 2.8832770769

C -4.8875103904 0.4927988245 2.2444725512

C -6.0465373232 0.6624914849 2.9830093535

C -6.0238108991 0.5755532455 4.3656642259

C -4.8172503257 0.3075038577 5.0057401702

C -3.6538075426 0.1368752348 4.2738423021

O -4.7238697444 0.2048086382 6.356080918

C -2.9597377434 3.5389589636 3.1635297835

C -1.8496144154 3.3702712097 3.9785738515

C -0.5784693123 3.3301452668 3.421736334

C -0.4162734744 3.4558783243 2.0491686901

C -1.5267422151 3.6213305518 1.2340393525

C -2.7979417864 3.662843798 1.7912373804

H -2.7372137198 -0.0661080417 4.8109489233

H   -4.9150771626   0.5742046766   1.1651743865

H   -6.9311939356   0.7100800987   4.9433775419

H   -6.9823055595   0.8680893558   2.4788377709

H   -2.6360686028   0.1607194979   0.9936475646

H   -1.014209951   0.0222119049   3.5839914797

H   1.5088506547   -0.0241985499   3.2304826315

C   2.4233755866   -0.0341351746   -1.4194696977

H   3.3175782546   -0.0431995587   1.3205139891

H   -5.5884752356   0.3324777677   6.7530962277

H   -3.9522100725   3.5543127092   3.5958619277

H   0.5739920814   3.408899818   1.6138878927

H   0.2871825333   3.1912304469   4.0574204111

H   -1.9766726076   3.2622750936   5.048539736

H   -3.6650864719   3.7830071701   1.1539679294

H   -1.4021389381   3.709877595   0.1620736362

H   2.16647175   -0.9155713205   -2.0100541886

H   2.1781857646   0.8463028405   -2.0164611484

H   3.500256961   -0.0410144276   -1.2537431068

4+benzene

C   0.027390002   2.1650131419   1.5777943929

C   -1.3575243214   2.077692968   1.5786483291

C   -2.0738358706   2.3762073424   0.428066453

C   -1.4036759936   2.7620199512   -0.7238443034

C   -0.018020408   2.8479766812   -0.7257051357

C   0.6972721365   2.5506413101   0.4256218329

C   -0.6412807685   -0.9557429257   -0.2966924468

C   -1.4522377401   -0.7011664476   -1.4018040719

C   -2.8351075195   -0.7433211946   -1.3652644123

C   -3.4589923651   -1.0537147525   -0.1617628428

C -2.6868002623 -1.3272282723 0.9627037523

C -1.30611275 -1.2799131115 0.9007916329

C 0.8015833574 -0.8269712546 -0.4661056599

C 1.7870507935 -0.9890175057 0.4247279914

C 3.1966174929 -0.7742950236 0.152372881

S 3.8139254959 -0.2213956481 -1.3646317344

C 5.4303562893 -0.2475124001 -0.7996681437

C 5.500432348 -0.6575869738 0.4941419668

C 4.225038381 -0.9567091762 1.0376726075

O -4.794709083 -1.1118175905 0.0155765707

C -5.6285703795 -0.834331856 -1.0909085798

O -0.565468582 -1.542974915 2.0027585257

H -3.1845736172 -1.5707106739 1.8938811292

H -0.9696492505 -0.4420297886 -2.3366147741

H -3.4013119487 -0.5260157962 -2.2581234796

H 1.0776503224 -0.528345225 -1.4745767124

H 1.5663752802 -1.2817622698 1.44099745

H 4.0625950268 -1.2987885477 2.0505302481

H 6.2376273764 0.0414808929 -1.453844766

H 6.428099067 -0.7441781774 1.0415180015

H -1.8800221072 1.7663729874 2.4744443511

H 0.5057940921 3.1422098801 -1.6266622839

H -1.9613391725 2.9869260246 -1.6244114416

H -3.1535287945 2.2966196521 0.4269534741

H 0.5867681181 1.9190262406 2.471447624

H 1.7786133663 2.605083377 0.4210839072

H -1.1454787537 -1.740320789 2.7421197197

H -6.6502366406 -0.9288284683 -0.7313507641

H -5.4721547989 0.1814987156 -1.4629614028

H -5.4664181674 -1.5490093955 -1.9017022647

5+benzene

C 3.1873359148 -1.0589417357 1.2848406848

C 2.2541994635 -0.8118223011 0.3176034712

S 2.9947063829 0.0049994374 -1.0175258712

C 4.5488412359 0.0038816216 -0.2688601432

C 4.4869666322 -0.5970463819 0.9504776943

C 0.8453263967 -1.146791884 0.3830142082

C -0.0499247859 -0.8915509794 -0.5784135705

C -1.4934229078 -1.1134620222 -0.5869080578

C -2.2445132022 -1.6451103181 0.4737533357

C -3.6258165672 -1.7620559222 0.3790757224

C -4.2908304172 -1.363376634 -0.7671415549

C -3.5734177403 -0.844354522 -1.8344867819

C -2.2001141426 -0.7274318746 -1.7307772819

O -1.59842082 -2.0392008137 1.5979941521

C -2.4549550504 1.6711591643 1.4919181024

C -2.9482045217 2.1436803004 0.2840248051

C -2.081972956 2.6965875714 -0.6485425776

C -0.7237578718 2.7789266001 -0.3724885897

C -0.2318279381 2.3092007918 0.8371477021

C -1.0973003545 1.7536435984 1.7682974581

H -1.6385539394 -0.3072119001 -2.5566219702

H -4.1795455635 -2.1703459438 1.2178606214

H -5.3674511369 -1.460716528 -0.8223271654

H -4.0798408814 -0.5282967574 -2.7365982159

H 0.307472112 -0.4186072944 -1.4895320841

H 0.5388387184 -1.6121131166 1.3086899295

H 2.9417018451 -1.559891986 2.2114089319

C 5.735689417 0.6044783279 -0.9465564939

H 5.3507560237 -0.7052326016 1.5922134152

H -2.235344335 -2.3736990157 2.2338709158

H -0.0468108862 3.2047622577 -1.1025983415

H 0.8287229688 2.3619610597 1.0484874705

H -0.7109210242 1.3737379028 2.7055221803

H -3.1290491328 1.2296609126 2.2150575378

H -4.00544381 2.0683155738 0.0640992811

H -2.465000818 3.05687149 -1.595125666

H 6.6107216644 0.5121196845 -0.3039069953

H 5.9559892478 0.1042574422 -1.8915783258

H 5.5840977798 1.6640567959 -1.1611569332

6+benzene

C -0.2673410745 -0.2227488995 0.1139702649

C -0.3015855038 -0.2975257737 1.5136864596

C 0.939775 -0.3088404694 2.1616917819

C 2.1345344663 -0.2422739703 1.4516438305

C 2.119945351 -0.1516962116 0.0679665359

C 0.9004247536 -0.1486747197 -0.6088042566

C -1.6070719999 -0.3210758814 2.1656045479

C -1.9133247175 -0.3345892429 3.4700634926

C -3.2585421704 -0.3376898617 4.0137693727

C -3.5923230467 -0.3479685049 5.3387596839

C -4.9927924116 -0.3474512039 5.5720421069

C -5.730542737 -0.3366330412 4.4291997337

S -4.6961100917 -0.326522861 3.0479215527

C -7.2164089643 -0.3335910631 4.2841792761

O 0.967480941 -0.3809768966 3.5132400496

O 3.2297474893 -0.0581593289 -0.6908901957

C 4.4871994067 -0.0792549358 -0.0487917836

H -1.2111140237 -0.2124520751 -0.4186245059

H 3.0654489047 -0.2375847371 2.0021385891

H 0.8906385681 -0.0815595023 -1.6879386671

H -2.4308786848 -0.3135703209 1.4554370816

H -1.1311511816 -0.3427771167 4.2158109629

H -2.8478256357 -0.3568220157 6.1232794491

H -5.4416588904 -0.3559307181 6.5560271783

H 1.8732647016 -0.302617721 3.8217080268

H -7.569162878 -1.2049976863 3.729262131

H -7.6810169315 -0.3506673195 5.2696658588

H -7.5688697472 0.5567177211 3.759799366

H 5.2315674989 0.0190424322 -0.8351836799

H 4.592540757 0.7535998054 0.6506940441

H 4.6476570555 -1.0231702087 0.4794560355

C 0.7694645727 3.1379428884 1.2568515986

C 0.9001251826 3.0100740335 2.6321994524

C 2.1599539126 3.0334965057 3.2141878637

C 3.2888846641 3.1858839309 2.4202403719

C 3.1571152919 3.315096424 1.0440825177

C 1.8971928078 3.2896999801 0.4627276164

H 0.0200069219 2.8775706721 3.2484202185

H 2.2614437874 2.9326125263 4.2877282344

H 4.2719985396 3.2068492741 2.8744348513

H 4.0365777476 3.432212737 0.423272149

H 1.7946237305 3.3775480897 -0.6113408004

H -0.2123538637 3.1051238269 0.8026006959

benzene+benzene

C -0.1251178599 0.9721976605 -0.0643976162

C -0.176211315 0.8547357628 1.3182082471

C 0.9672855869 0.5169073764 2.0281030977

C 2.1613822762 0.2938192668 1.3566274858

C 2.2128245623 0.4137786589 -0.0242732743

C 1.0697917252 0.7528541481 -0.7353109353

C 1.7210351237 -3.0525834553 -0.571313243

C 0.5413839182 -2.8289305192 0.1249470201

C 0.5173735673 -2.954597117 1.506131583

C 1.673553199 -3.29932997 2.1927088278

C 2.8541079879 -3.5192164597 1.496986303

C 2.8776537816 -3.396553916 0.114098838

H -0.3574886765 -2.5479637845 -0.408645113

H 1.7407549418 -2.948989355 -1.6489096383

H 3.7988258945 -3.5668999514 -0.4290138846

H 3.7567460156 -3.7858231192 2.0325157452

H 1.6551606376 -3.3927437324 3.2713366997

H -0.4013151705 -2.7731071234 2.0492527116

H 3.0501713284 0.0177489447 1.9093317883

H 0.9261791053 0.4176833127 3.1055128714

H -1.1086321298 1.0245731287 1.8419602426

H -1.0175347879 1.2342371928 -0.6189726827

H 1.1096422723 0.8419986424 -1.8137212956

H 3.1427105001 0.2325662122 -0.5480886392

Geometries of H-bonded complexes between molecules 2-6 and CH_3OH, optimized in diethyl ether solution utilizing SMD-ωB97XD/6-311+G(3df,2p) model.

2-CH3OH

C -0.1198585708 -0.1926494031 -0.0565302371

C -0.0961166876 0.0830910493 1.3238939712

C 1.1512241998 0.3387632966 1.9057537347

C 2.3283944317 0.3274277866 1.1810708413

C 2.2800108492 0.0499056091 -0.1776429121

C 1.0660314578 -0.209088481 -0.7886720104

C -1.2676295517 0.1273661198 2.19711804

C -2.557194618 -0.0865172886 1.9064327308

C -3.6506207667 -0.0152498428 2.8592850294

S -3.4551535623 0.3713858161 4.5323102585

C -5.1335533917 0.2290503688 4.8358345048

C -5.8199657911 -0.0995109449 3.7095440146

C -4.9725938442 -0.2386752163 2.5813330324

O -1.3003593666 -0.4401311991 -0.6524513872

O -1.0661011731 -0.495669653 -3.3728188768

C -1.040482124 0.8567107022 -3.818890592

H 1.1833237667 0.5538754948 2.967521937

H 1.0247825549 -0.4367880805 -1.8468150084

H 3.1883175338 0.0308032867 -0.7664886557

H 3.2716440061 0.5307619949 1.6700320872

H -1.015697526 0.371736781 3.2265429424

H -2.8550506994 -0.3348288368 0.8980440461

H -5.3194166152 -0.4965765365 1.5901243254

H -5.5197953155 0.395405542 5.8290246053

H -6.8913200853 -0.2378015715 3.6843811723

H -1.1894588018 -0.5377430022 -1.617667278

H -0.7405003539 0.9178367544 -4.8666506848

H -2.0114022836 1.3388662292 -3.6872746004

H -0.3040444606 1.3791251424 -3.2108729767

H -1.7237683502 -0.981505185 -3.873494686

3-CH3OH

C 0.031023999 0.2527318831 0.0593198504

C -0.0179934556 -0.0204281344 1.4001412135

S 1.5260981114 -0.5613490251 1.9534456337

C 2.2142323661 -0.4160977209 0.3936676358

C 1.3064234895 0.0269734547 -0.5162093514

C -1.1825008369 0.1159350184 2.2541702477

C -1.2375108856 -0.1461432491 3.5627475459

C -2.4096025105 -0.0114281813 4.4348337735

C -3.6465670923 0.4393574531 3.9745105469

C -4.7288392378 0.5492410016 4.8370189829

C -4.585131103 0.2099834255 6.1833897845

C -3.3581144413 -0.2390370655 6.6418208881

C -2.2763758706 -0.3516472164 5.7833647844

O -5.8946857229 0.9909702939 4.3262369014

O -7.935705158 0.753324522 6.144266712

H -3.7946137296 0.7144201646 2.9385604674

H -1.3219659321 -0.7030017713 6.1548913599

H -5.4259376134 0.3076845658 6.8586837123

H -3.2452261925 -0.501901153 7.6862002606

H -0.3416391521 -0.4984659283 4.0670092361

H -2.0663471952 0.4669772355 1.7316954793

H -0.8293031428 0.6064806326 -0.4920023173

H 3.251233455 -0.6660671274 0.2342417969

H 1.5380262891 0.1870128998 -1.5593340746

H -6.5995927182 0.9684262526 4.9996681493

C -8.3753505919 -0.6008264404 6.1375998749

H -8.6928781328 1.3312562621 6.0353598109

H -9.0454929569 -0.7993735393 6.9762134218

H -8.8768497944 -0.8535608465 5.2009893717

H -7.4892396728 -1.2246994771 6.2401205827

4-CH3OH

C 0.0127875035 -0.0383966878 0.0327768486

C 0.0109145802 0.0047358128 1.4438527251

C 1.2573912264 0.0374765467 2.068903923

C 2.4598394062 0.0309080575 1.3823405397

C 2.4290195228 -0.0076731781 -0.0084384049

C 1.207202455 -0.0411689636 -0.6723285348

C -1.1779800628 0.0162911982 2.2908945131

C -2.4729041152 0.0054836571 1.9479909716

C -3.5853309592 0.0210640844 2.8814212198

S -3.4091529092 0.0440867448 4.6007883128

C -5.1036410096 0.0532879363 4.8482367042

C -5.7803106648 0.0388042334 3.6696607092

C -4.9133249921 0.020166658 2.5474968497

O 3.5246593858 -0.0139685874 -0.7960582181

C 4.7936952874 0.0413789357 -0.1784715745

O -1.1636553652 -0.0751888943 -0.6150485917

O -0.8481045486 -0.5290469217 -3.2830899536

C -0.6246403522 -1.9209923371 -3.4847389108

H -5.5051358639 0.0686933872 5.8490604111

H 1.1978329269 -0.0576024726 -1.754703629

H 3.3900292103 0.0573210037 1.9292828834

H -0.9375575317 0.0399437436 3.3516094973

H -2.7604496895 -0.0149573262 0.9067447959

H -5.2510752696 0.0071126201 1.5203429044

H -6.858829729 0.04145528 3.6024328928

H -1.0310983989 -0.1683289723 -1.5784039132

H 5.5233120073 0.0365798147 -0.9846835802

H 4.9131254717 0.9567351068 0.4068569932

H 4.964239147 -0.8267875507 0.4633298785

H 1.2837499359 0.068795228 3.1519995426

H -1.536995767 -0.2290353214 -3.8785307185

H -0.2635574982 -2.116541036 -4.4960747993

H 0.1394485062 -2.2280584668 -2.7729111519

H -1.531108641 -2.501373752 -3.3006659184

5-CH3OH

C 0.0879755802 0.3254019137 0.1608105985

C 0.0239801717 0.0125454231 1.5238204152

C 1.2375606264 -0.2777523199 2.1760059048

C 2.4379097812 -0.25257084 1.4682273288

C 2.4604638332 0.0627213831 0.1211652557

C 1.2777919979 0.3556055832 -0.5427033312

C -1.2948375011 0.0181962476 2.1548191379

C -1.635583129 -0.2475821882 3.4227570468

C -2.9862011086 -0.2088192486 3.9506966313

C -3.3548072476 -0.4749497502 5.2393375689

C -4.7505281375 -0.3547202466 5.4669824814

C -5.4495922718 0.0024964816 4.3556146479

S -4.3846260226 0.1938550207 3.0121589709

C -6.9197226856 0.2215654743 4.2159685861

O 1.2177628556 -0.5799145124 3.4871085384

O 3.7328401083 -0.6357192958 4.5526894006

C 4.1147035727 0.7172915195 4.7811335003

H -0.8386789764 0.5526255323 -0.3534312642

H 3.3573199159 -0.4924599114 1.9883013884

H 3.404812139 0.0753822785 -0.4081470455

H 1.282211278 0.602965455 -1.5958017938

H -2.0915002107 0.2812972629 1.4628018698

H -0.8784688769 -0.5156089709 4.1456715179

H -2.6379959458 -0.7497680394 6.0012085934

H -5.2244242091 -0.5268218161 6.4238193179

H 2.1244369796 -0.6785458673 3.8354386768

H -7.3615112065 -0.460676907 3.4871936741

H -7.4085371253 0.0533708646 5.1751463708

H -7.146872049 1.2399802253 3.894938048

H 5.1782734766 0.7897331342 5.0158603132

H 3.9196013273 1.2637099022 3.860097077

H 3.5304673927 1.1662742043 5.587073091

H 3.8711315627 -1.1427915154 5.3545418207

6-CH3OH

S 0.0468301749 0.0148616594 -0.1946721973

C 0.1278028923 -0.1148837335 1.5306204821

C 1.4304509482 -0.2418717948 1.923276599

C 2.352903468 -0.2351322091 0.8438830017

C 1.759309946 -0.1029607645 -0.3729533116

C -1.0303282004 -0.0886247144 2.404438031

C -2.3021244077 0.0334868905 2.0000731156

C -3.5308638813 0.075779197 2.7872694272

C -4.7439500675 0.2227715113 2.0999278226

C -5.9668286373 0.2849768706 2.7283466667

C -6.0160850379 0.1963877905 4.1192869103

C -4.8415536458 0.0426006306 4.8407103045

C -3.6098140969 -0.0142919452 4.1863067169

O -2.4774974003 -0.1555002759 4.8950302669

O -7.2391749617 0.2682917335 4.6853627825

C -7.3323595816 0.2178373195 6.0926702994

C 2.4033591486 -0.0541730587 -1.7189513246

O -2.8097519135 -0.2211363891 7.6094134765

C -3.6997224844 0.6441367144 8.3024314258

H -4.71032273 0.2936441561 1.0189144211

H -4.8572395725 -0.0398093999 5.9171762952

H -6.8835570621 0.4025704559 2.1672411695

H -2.4851353988 0.1191852253 0.9311463325

H -0.7993622684 -0.1764847994 3.4564714029

H 1.7145767323 -0.3376800883 2.9624457468

H 3.4240741082 -0.3254174573 0.9637714845

H -2.6588292314 -0.1910658789 5.8527542937

H 2.2006078987 0.8895695945 -2.2290834592

H 3.4831852872 -0.1545738392 -1.6124899714

H 2.0510898689 -0.8608240697 -2.3647301155

H -8.3892264494 0.3096960031 6.3308859636

H -6.9602298965 -0.7328123277 6.484602656

H -6.7876967138 1.044126143 6.5581041984

H -3.3068249304 0.9033493731 9.287609604

H -3.7885646725 1.5532308515 7.7108756627

H -4.6892725341 0.1947086083 8.4168889894

H -2.7491241358 -1.0572574579 8.0739162573

phenol-CH3OH

C -0.2265483086 0.1277568331 0.1322156021

C -0.075378815 0.5123967133 1.4636151468

C 1.1596990131 0.3565981846 2.0890316423

C 2.2259151263 -0.1810385684 1.387670309

C 2.0819381562 -0.5707961919 0.0619790435

C 0.8503247559 -0.4112462764 -0.5561935526

O -1.0852580595 1.0389399646 2.183668827

O -3.4648662568 0.8730472214 0.822642708

H 1.2676955227 0.6597438004 3.1224722758

H 3.1811489619 -0.2971430639 1.8847866609

H 2.9183968779 -0.9903551011 -0.4808249415

H 0.7212688773 -0.7056818957 -1.590498234

H -1.1821316056 0.2597321917 -0.3603902763

H -1.9131429003 1.0427513736 1.6687063582

C -3.9606929087 -0.4521771577 0.9800124852

H -4.1552322103 1.4974814135 1.052026546

H -4.7903190357 -0.6442631241 0.2967975697

H -3.1432329227 -1.1295982396 0.7397122534

H -4.2820235526 -0.6394389971 2.0068342547

Geometries of molecules 2-6 optimized in the gas phase utilizing ωB97XD/6-311+G(3df,2p) model.

2-deprot

S 0.0093915292 0.0058974646 0.0117206746

C -0.0192591864 -0.000499885 1.7468065785

C 1.2695995954 -0.0034832033 2.221283057

C 2.2577392373 -0.0008863933 1.2045528627

C 1.7265151534 0.0039151838 -0.0466558625

C -1.2313962513 -0.0021174229 2.5336414005

C -2.4793571795 0.0019319949 2.0236282117

C -3.7383606116 0.0007155291 2.7246605109

C -4.912776822 0.0058590767 1.9582627771

C -6.1758016247 0.0052162566 2.5116585764

C -6.2802623396 -0.0010119716 3.9119871516

C -5.1643109017 -0.0062050354 4.7068522115

C -3.8223907784 -0.0055416501 4.1773824335

O -2.8204741745 -0.0107728909 4.9319655373

H -4.8100281638 0.0105532325 0.8755682401

H -5.2516668699 -0.0110768988 5.7876902554

H -7.2634229429 -0.0017323505 4.3742615784

H -7.0591114208 0.0093058144 1.8857375332

H -2.5782049546 0.0068566857 0.9365353197

H -1.1177398284 -0.0068501865 3.6102365218

H 1.484978907 -0.0074789538 3.2802551075

H 2.2419705065 0.006609092 -0.9927993112

H 3.3218738796 -0.0026934487 1.3973817441

2-prot

S 0.0137807728 0.0060015193 0.0081684643

C -0.0066749448 -0.0008991253 1.733374729

C 1.2730792042 -0.0040224244 2.2192834412

C 2.2621356163 -0.000935845 1.2053892559

C 1.7225782386 0.0044751014 -0.0423795775

C -1.2186715421 -0.0028929366 2.5311320396

C -2.4628067083 0.0009795629 2.0393669308

C -3.7423233518 -0.0003370119 2.7453841107

C -4.9093818839 0.0064053493 1.9743303407

C -6.1743710753 0.0058465315 2.5313781197

C -6.3053842803 -0.0017107698 3.9107058158

C -5.1736303966 -0.0085361204 4.7067000453

C -3.9059522285 -0.0079385961 4.1397214177

O -2.7991169489 -0.0146504244 4.9263308936

H -4.8050788077 0.0122728246 0.8960704475

H -5.2696395027 -0.0144153618 5.7871153828

H -7.2842764799 -0.0023094155 4.371236552

H -7.0487108037 0.0112343107 1.8955296151

H -2.57658435 0.00607442 0.9582780856

H -1.0547664216 -0.0078763866 3.5987224303

H 1.4895076738 -0.0084345091 3.278154303

H 2.2360122533 0.0076898458 -0.9895177613

H 3.3252400893 -0.0027075987 1.3947904697

H -3.0609896996 -0.0207275619 5.8473141045

3-deprot

C -0.0245089377 0.028622569 0.022277536

C 0.0301153027 -0.0038986791 1.4234689905

C 1.2567787101 -0.0356711159 2.0746518972

C 2.5144094266 -0.0374101262 1.3830084412

C 2.3922271548 -0.0032164064 -0.0534191323

C 1.1718380589 0.0281877932 -0.692021713

C -1.2455113756 -0.0024879387 2.1414984267

C -1.4450325232 -0.0298708088 3.4646061142

C -2.7314938992 -0.027157252 4.1321636541

C -2.9578136518 -0.0537187054 5.481982643

C -4.3328475243 -0.0427859669 5.832913761

C -5.1633350047 -0.0078890979 4.7564200627

S -4.2462383152 0.0116343485 3.2981811023

O 3.6242662941 -0.0661024716 1.9730725063

H 1.3099041861 -0.0609606739 3.1574212265

H -0.9804063082 0.0534581049 -0.4877255856

H 3.3141330879 -0.0030735449 -0.6253155239

H 1.1409554934 0.0531982528 -1.7777909483

H -2.1221664686 0.0244638972 1.4958106139

H -0.5937468242 -0.0569774684 4.1359381632

H -2.1489543141 -0.0803610316 6.1987859882

C -6.6609361556 0.0127047967 4.731819013

H -4.6938780163 -0.0602497682 6.8529727722

H -7.0425003503 0.0425936843 3.7114330211

H -7.075763946 -0.8758575135 5.2117044585

H -7.0521155792 0.8877110634 5.2545371477

3

C 0.0152702033 0.0188287253 0.0055161532

C 0.0096163996 0.0451958626 1.4020834107

C 1.2295002196 0.0621350909 2.076091439

C 2.4217501445 0.0376350881 1.372581039

C 2.420649816 0.0026102097 -0.0178822308

C 1.2105708212 -0.0045644238 -0.6912063387

C -1.2797332553 0.0561046471 2.09881351

C -1.4765824343 -0.1063098642 3.4093280214

C -2.7530077527 -0.0968962871 4.0932111534

C -2.9549051049 -0.2876705897 5.430901402

C -4.3194394761 -0.2250456185 5.8095077169

C -5.1565439078 0.0136804004 4.7637135894

S -4.2648239221 0.1644611079 3.298783837

O 3.571140462 0.0556184516 2.0968443445

H 1.278491334 0.1068007024 3.1552836016

H -0.9242465673 0.0112543683 -0.5317577423

H 3.3560554492 -0.0132247189 -0.5651708053

H 1.203547797 -0.0282352623 -1.7730761672

H -2.1431593682 0.1972679875 1.4550095334

H -0.629336205 -0.2732789739 4.0664542876

H -2.1423069431 -0.4686180386 6.1205421939

C -6.6444325817 0.1441268234 4.7831844656

H -4.6724243584 -0.3521367922 6.8232364099

H 4.3262786682 0.0440995244 1.5086644821

H -7.1230689156 -0.6086640959 4.1548911854

H -7.010342279 0.0152830959 5.8009555674

H -6.9675186814 1.1247191091 4.4301879724

4-deprot

C -0.0096084431 0.0024728343 -0.0093555104

C -0.0281125419 0.007110407 1.384982587

C 1.2360653181 0.0107757986 2.1154888615

C 2.4147904158 0.0091358454 1.2951646514

C 2.370586074 0.0044857234 -0.0758969869

C 1.1475137699 0.0010177324 -0.7679250115

C -1.3092966271 0.0080865451 2.0438100587

C -1.5779673154 0.0121997621 3.3645474344

C -2.9050281521 0.012809839 3.9364112627

C -3.2249015427 0.0183790071 5.2720045426

C -4.6189681836 0.0175525208 5.5305582664

C -5.3658109385 0.0113424632 4.3948634457

S -4.3649851575 0.0062619011 2.9982910227

O 1.3070118495 0.0149818085 3.366327579

O 3.5861870762 0.0033838812 -0.7205667892

C 3.5906109713 -0.000078438 -2.1165618562

H -6.4389945721 0.0093953219 4.2992374195

H 3.3738758304 0.0117288861 1.7974715385

H 1.0911752864 -0.0026977243 -1.8457985606

H -2.1587080261 0.0050565366 1.3582135401

H -0.7459095675 0.0154208628 4.0569460197

H -2.4630867935 0.0229994232 6.0384482749

H -5.0484649424 0.0214836065 6.5230803165

H 4.6361425957 0.0003305913 -2.4234422218

H 3.1013023249 -0.8909518509 -2.5266068166

H 3.0992756548 0.8876347209 -2.5309967009

H -0.963681215 -0.0001795306 -0.5302457226

4

C -0.0037763995 0.003098968 -0.0009207735

C -0.0151891225 0.0068467239 1.3924453927

C 1.243831324 0.0090904502 2.0213959028

C 2.4133087975 0.0076268216 1.2842120314

C 2.3794506666 0.00386071 -0.1060602211

C 1.1541516476 0.0015580219 -0.7607434691

C -1.308650453 0.0080980155 2.06944044

C -1.5884186552 0.0115809597 3.3777391316

C -2.9244049359 0.0124979252 3.9448618251

C -3.2302460799 0.0165523965 5.2790117646

C -4.6228407319 0.0164927159 5.5400662714

C -5.3627365211 0.0123696064 4.3999175273

S -4.3721859522 0.0085567889 3.0056839598

O 1.2903350609 0.0127675614 3.3766167813

O 3.5817634454 0.0027295479 -0.721348592

C 3.6092392574 -0.0009964168 -2.1290425937

H -6.4356125285 0.0113494383 4.3020781061

H 3.3773066577 0.0094014905 1.7788991453

H 1.0867982695 -0.0013775948 -1.8376588008

H -2.1469095694 0.0057969162 1.3769284299

H -0.7904796832 0.0140105897 4.1058815625

H -2.469196622 0.0194934564 6.046402883

H -5.0543274039 0.0193743319 6.5300469846

H 2.202877754 0.0137453824 3.666766677

H 4.6582356405 -0.0012738713 -2.4120904062

H 3.1263716947 -0.8941793451 -2.5345265015

H 3.125563536 0.8895828142 -2.5392627389

H -0.9565969741 0.0012890383 -0.5163319186

5-deprot

C -0.0032931915 -0.0000930098 0.0151049146

C -0.0116049114 -0.0001349591 1.3850075757

S 1.6285130769 0.0004166866 1.9637466664

C 2.2959411669 -0.0003833231 0.3695643365

C 1.2987480005 -0.0001517405 -0.5531609557

C -1.1689291204 -0.0002820433 2.2500221119

C -1.1229729031 0.0000263356 3.5971725137

C -2.217195745 -0.0001107875 4.5359852847

C -3.6092722756 -0.0006461343 4.112260814

C -4.5700997693 -0.0006562841 5.1880792458

C -4.2106740222 -0.0002566863 6.5104602435

C -2.8609735693 0.000218353 6.8967940793

C -1.9046891463 0.000289429 5.9027776475

O -3.9708052625 -0.0009220675 2.9109623634

C 3.7723205805 -0.0007691753 0.1402832056

H -0.8532881932 0.0006696897 6.181036344

H -5.6144339191 -0.0010068577 4.8960242019

H -4.9846894396 -0.0003101872 7.2728652755

H -2.5789693166 0.0005372267 7.9420519157

H -0.137413106 0.0004374085 4.0664068364

H -2.1393069532 -0.0006583501 1.7699691502

H -0.9186055942 -0.0001754346 -0.5595791901

H 1.4919707146 -0.0003023121 -1.6185860873

H 4.2538976275 -0.8812735267 0.5726425411

H 3.9765987466 -0.0014628385 -0.9311245926

H 4.2541456417 0.8801345293 0.571549647

5

C	0.0013059118	-0.0003471977	0.0133248473
C	0.0078531864	-0.0003558379	1.3794403987
S	1.6360978694	-0.0002089451	1.9599399173
C	2.294404389	-0.000411992	0.3688081998
C	1.2988816659	-0.0003791061	-0.5584238842
C	-1.1590478351	-0.0003966726	2.2394004872
C	-1.1295959682	-0.0001888862	3.5773586186
C	-2.2358127911	-0.0001972838	4.5322890909
C	-3.6004648367	-0.000597282	4.2024970614
C	-4.5721854481	-0.0005593416	5.1945456845
C	-4.2189898198	-0.0001476748	6.5324762198
C	-2.8797572661	0.0002440832	6.887849406
C	-1.9183663778	0.0002171763	5.8945948709
O	-3.9548357211	-0.0010067743	2.8913309152
C	3.7705883847	-0.0004664526	0.1401526209
H	-0.8709074277	0.0005231833	6.1711307552
H	-5.6187997248	-0.0008438325	4.9094755221
H	-4.9906813026	-0.0001323122	7.2906486356
H	-2.5864267942	0.0005673206	7.9284537236
H	-0.1550168273	0.0000440823	4.0589145438
H	-2.1037774872	-0.0005988403	1.7153242413
H	-0.9143333446	-0.0003784396	-0.5612360497
H	1.4912537508	-0.0004379374	-1.6222158267
H	-4.9094603638	-0.0015139359	2.8180048116
H	4.2458624119	-0.8811581484	0.5750500806
H	3.9762887353	-0.0009784162	-0.9295093188
H	4.2457718589	0.8807015895	0.5741880137

6-deprot

C 0.0001359318 -0.009017509 0.0024435174

C -0.0208282236 -0.0034094467 1.4033986817

C 1.2388965887 0.0047158634 2.1285737281

C 2.4300099332 0.0062318641 1.3106690197

C 2.3807707722 0.0004354092 -0.0595617666

C 1.1543746504 -0.0073607429 -0.7466194391

C -1.2997409043 -0.0059580281 2.0624570855

C -1.5728705151 -0.0017322473 3.3835709074

C -2.9010945664 -0.004928429 3.95017074

C -3.2351252843 0.0006186255 5.2792708297

C -4.6349512712 -0.0046182241 5.5220174677

C -5.385115954 -0.0142537257 4.3895044047

S -4.3578668296 -0.0168596793 2.9989568165

C -6.8736266871 -0.0223425536 4.26162748

O 1.3217622794 0.0102384091 3.3783440555

O 3.4927884884 0.0016919687 -0.8648893941

C 4.7443229842 0.0095868192 -0.2428416463

H -0.9541498798 -0.0150360842 -0.5180075318

H 3.3643770034 0.0122524269 1.8543001996

H 1.1428023328 -0.0118039772 -1.8276239482

H -2.1488486022 -0.0121778186 1.376519433

H -0.7449434799 0.004622068 4.0807214648

H -2.4828517259 0.0083667411 6.0552469592

H -5.0754101276 -0.001340981 6.5112646433

H -7.2351541575 -0.9088057331 3.7345485084

H -7.3264377243 -0.0169798883 5.2539375343

H -7.2435665057 0.8525086116 3.7211890088

H 5.4872164656 0.0095276662 -1.0398703792

H 4.8832461418 0.9007667702 0.3786926749

H 4.8908687143 -0.8752883586 0.385911156

6

C 0.0126631569 -0.0091078189 0.0113152827

C -0.0032310156 -0.0041960766 1.4127401614

C 1.246589909 0.0028154422 2.0407088755

C 2.4315478594 0.0047503106 1.3088270455

C 2.3975776389 -0.0002909958 -0.0784484989

C 1.1688465637 -0.0073156928 -0.7335104285

C -1.2995862081 -0.0067895064 2.0841356701

C -1.5863650269 -0.0031045354 3.3914748074

C -2.9239431575 -0.0062453104 3.9515815755

C -3.2422534786 -0.0025734992 5.2799580446

C -4.6388764326 -0.0070888888 5.5277375479

C -5.386457749 -0.014196046 4.3916344746

S -4.3687401 -0.0153317891 3.0017284097

C -6.8748420206 -0.0205908274 4.2642839021

O 1.2936890557 0.0077905662 3.3970018474

O 3.4975272493 0.0011459646 -0.8623888355

C 4.7623472695 0.0080837653 -0.2481897181

H -0.9389022877 -0.0145910779 -0.5063193842

H 3.3724478811 0.0102298428 1.8432032015

H 1.1460042583 -0.0112312005 -1.813720224

H -2.1349409765 -0.0125906538 1.3882475219

H -0.7925241042 0.0026665033 4.1243354975

H -2.4890159534 0.0032242577 6.0552813654

H -5.0781705056 -0.0051451207 6.5155878462

H 2.2050363436 0.0124586647 3.6897501997

H -7.2306901316 -0.9053408119 3.7338377318

H -7.3275201509 -0.0178158844 5.255070637

H -7.237594865 0.8564249258 3.725744011

H 5.4920640572 0.0079897336 -1.0531810017

H 4.9045400363 0.9034499819 0.364033125

H 4.9112671663 -0.8818356621 0.3703424103

phenol-deprot

C 0.004727735 0.0011513928 0.0001537778

C -0.0224281454 -0.000145258 1.4399785603

C 1.285245229 0.0001666357 2.0434423378

C 2.4484684798 0.0000782227 1.3001825276

C 2.4281958136 0.0002388739 -0.0956352909

C 1.1809485033 0.0011265825 -0.7224809192

O -1.0900927042 0.0013235531 2.109037333

H 1.3261949781 0.0003207379 3.1281433384

H 3.4048451937 -0.0000775706 1.8179412458

H 3.3461682884 0.0000516215 -0.6708426496

H 1.1319720575 0.0015581927 -1.8089008998

H -0.9534746041 0.0015896151 -0.5098851281

phenol

C 0.0047216661 -0.0001851079 0.0024540269

C 0.0048847404 0.0000025239 1.3917212798

C 1.2063575875 -0.0002257054 2.0895168604

C 2.4007588445 -0.0006405346 1.3905790806

C 2.4132351498 -0.0008329573 0.0020858414

C 1.2096179651 -0.0006032415 -0.6841735996

O -1.1414800155 0.0004136227 2.1205960299

H 1.1858002535 -0.0000746431 3.1706642073

H 3.3341184255 -0.0008168162 1.9386667679

H 3.3503438128 -0.0011580633 -0.5371151718

H	1.2020656317	-0.0007482897	-1.7664824211
H	-0.9338062443	-0.0000040375	-0.540405223
H	-1.8994592345	0.0004925706	1.535975092

Table S1: Bond distances (in Å) of neutral and deprotonated resveratrols 2 and 3 obtained by model M(II)

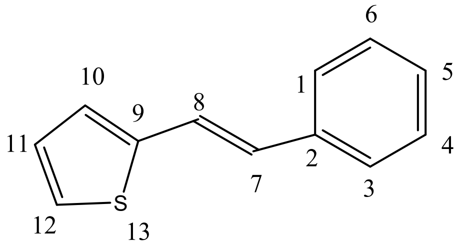

2-neutral		2-anion	
C(1)-C(2)	1.404	C(1)-C(2)	1.455
C(2)-C(3)	1.399	C(2)-C(3)	1.402
C(3)-C(4)	1.382	C(3)-C(4)	1.379
C(4)-C(5)	1.395	C(4)-C(5)	1.404
C(5)-C(6)	1.383	C(5)-C(6)	1.370
C(6-C(1)	1.389	C(6-C(1)	1.443
C(2)-C(7)	1.461	C(2)-C(7)	1.441
C(7)-C(8)	1.337	C(7)-C(8)	1.348
C(8)-C(9)	1.451	C(8)-C(9)	1.445
C(9)-C(10)	1.368	C(9)-C(10)	1.373
C(10)-C(11)	1.416	C(10)-C(11)	1.418
C(11)-C(12)	1.359	C(11)-C(12)	1.359
C(12)-C(13)	1.709	C(12)-C(13)	1.718
C(1)-O	1.358	C(1)-O	1.254
**3-neutral**		**3-anion**	
C(1)-C(2)	1.394	C(1)-C(2)	1.389
C(2)-C(3)	1.397	C(2)-C(3)	1.403
C(3)-C(4)	1.384	C(3)-C(4)	1.394
C(4)-C(5)	1.384	C(4)-C(5)	1.377
C(5)-C(6)	1.391	C(5)-C(6)	1.442
C(6-C(1)	1.384	C(6-C(1)	1.435
C(2)-C(7)	1.465	C(2)-C(7)	1.463
C(7)-C(8)	1.335	C(7)-C(8)	1.338
C(8)-C(9)	1.448	C(8)-C(9)	1.449
C(9)-C(10)	1.366	C(9)-C(10)	1.369
C(10)-C(11)	1.417	C(10)-C(11)	1.419
C(11)-C(12)	1.361	C(11)-C(12)	1.360
C(12)-C(13)	1.721	C(12)-C(13)	1.722
C(6)-O	1.359	C(6)-O	1.257
C(12)-CH_3	1.500	C(12)-CH_3	1.498
